# Pharmacological potential of *Withania somnifera* (L.) Dunal and *Tinospora cordifolia* (Willd.) Miers on the experimental models of COVID-19, T cell differentiation, and neutrophil functions

**DOI:** 10.3389/fimmu.2023.1138215

**Published:** 2023-03-07

**Authors:** Zaigham Abbas Rizvi, Prabhakar Babele, Upasna Madan, Srikanth Sadhu, Manas Ranjan Tripathy, Sandeep Goswami, Shailendra Mani, Madhu Dikshit, Amit Awasthi

**Affiliations:** ^1^ Immuno-biology Lab, Translational Health Science and Technology Institute, NCR-Biotech Science Cluster, Faridabad, Haryana, India; ^2^ Immunology-Core Lab, Translational Health Science and Technology Institute, NCR-Biotech Science Cluster, Faridabad, Haryana, India; ^3^ NCD, Translational Health Science and Technology Institute (THSTI), NCR Biotech Science Cluster, Faridabad, Haryana, India; ^4^ Pharmacology, CSIR-Central Drug Research Institute, Lucknow, Uttar Pradesh, India

**Keywords:** *Withania somnifera (Ashwagandha)*, *Tinospora cordifolia*, SARS-CoV-2, hamster model, T cells, neutrophils, and hACE2 transgenic mice, COVID-19

## Abstract

Cytokine release syndrome (CRS) due to severe acute respiratory coronavirus-2 (SARS-CoV-2) infection leads to life-threatening pneumonia which has been associated with coronavirus disease (COVID-19) pathologies. Centuries-old Asian traditional medicines such as *Withania somnifera* (L.) Dunal (WS) and *Tinospora cordifolia (*Willd.) Miers (TC) possess potent immunomodulatory effects and were used by the AYUSH ministry, in India during the COVID-19 pandemic. In the present study, we investigated WS and TC’s anti-viral and immunomodulatory efficacy at the human equivalent doses using suitable *in vitro* and *in vivo* models. While both WS and TC showed immuno-modulatory potential, WS showed robust protection against loss in body weight, viral load, and pulmonary pathology in the hamster model of SARS-CoV2. *In vitro* pretreatment of mice and human neutrophils with WS and TC had no adverse effect on PMA, calcium ionophore, and TRLM-induced ROS generation, phagocytosis, bactericidal activity, and NETs formation. Interestingly, WS significantly suppressed the pro-inflammatory cytokines-induced Th1, Th2, and Th17 differentiation. We also used hACE2 transgenic mice to further investigate the efficacy of WS against acute SARS-CoV2 infection. Prophylactic treatment of WS in the hACE2 mice model showed significant protection against body weight loss, inflammation, and the lung viral load. The results obtained indicate that WS promoted the immunosuppressive environment in the hamster and hACE2 transgenic mice models and limited the worsening of the disease by reducing inflammation, suggesting that WS might be useful against other acute viral infections. The present study thus provides pre-clinical efficacy data to demonstrate a robust protective effect of WS against COVID-19 through its broader immunomodulatory activity

## Introduction

The first reported case of SARS-CoV-2 was in 2019 and has since then become a predominant cause of global morbidity and mortality (1). COVID-19 was declared a pandemic by the World Health Organization (WHO) in March 2020 demanding the development of therapeutic interventions and vaccine candidates to mitigate COVID-19-related pathology and mortality (2–5). One of the hallmarks of severe COVID-19 is cytokine release syndrome (CRS) which is responsible for elevated pro-inflammatory cytokines in the pulmonary region leading to respiratory distress (6–9). Moreover, attenuated functionality in other major organs or multiple organ failure has also been manifested in a significant number of COVID-19 cases (10–12). Active vaccination strategy helped in alleviating the COVID-19 severity and related death, however, the continuous evolution of the ancestral virus using acquiring mutations led to immune evasion and poorer protection against variants of concern (VoC) and emerging variants of SARS-CoV-2 (13, 14). In addition, COVID-19 vaccines may not be sufficient to confer protection in immunocompromised individuals or the individuals with co-morbid conditions. Therapeutic antiviral drugs such as Remdesivir (RDV) and immunosuppression by Dexamethasone (DXM) were the most acceptable therapeutic options against SARS-CoV-2 infection. While DXM was effective in reducing the overall morbidity and mortality arising due to COVID-19 and showed success in clinical trials, a randomized clinical trial of RDV did not show any significant protection in COVID-19 deaths and was marginally successful in giving relief from clinical symptoms (15, 16).

In addition, a major issue with synthetic drugs is also their off-target reactivity limiting their usage in clinical cases. DXM, for example, is a broad immuno-suppressant drug and is known to suppress the overall immune response which may lead to a rise in opportunistic pathogenic infections and other life-threatening complications (17). The alternate strategy of COVID-19 management includes prophylaxis or preventative strategies with immunomodulators to improve immunity against SARS-CoV-2 infection. In this regard, plant-derived immunomodulators have gained considerable interest owing to their prolonged human use and better safety. The emerging line of evidence has shown that the use of herbal extracts from traditional medicine systems might help in mitigating the COVID-19 pathology (18–20). In the current study, we investigated the efficacy of WS (Ashwagandha), TC (Guduchi), and *Piper longum* L. (Thippali), in the *in vitro* cell-based systems and animal models. WS, a shrub traditionally used in India and other Asian countries, is known to possess immunomodulatory properties. Previous *in-vitro* and *in-vivo* studies have shown that WS could play a role in the regulation of inflammation by suppressing pro-inflammatory cytokines (21–24). More recently, in-silico docking studies showed the potent inhibitory potential of Withanone, an active ingredient of WS, against SARS-CoV-2 virulence proteins such as spike protein (22, 25). Other reports have shown Withaferin A, a bioactive steroidal lactone derived from WS, to be anti-inflammatory by reducing the levels of inflammatory cytokines such as IL-6 and TNFα which is desirable in COVID-19 patients to alleviate pulmonary pathology (26, 27). More recently, we have also characterized the anti-viral component of WS which was found to exhibit potent anti-viral property *in-vitro* (28). Similarly, TC and PL have also been shown to modulate inflammatory responses in various disease conditions. TC, particularly, was found to exert anti-viral activity against SARS-CoV-2 in *in-silico* and *in-vitro* studies (25). However, there is still a lack of evidence on the protective efficacy and immunomodulatory potential of these herbal extracts in the *in-vivo* models of COVID-19.

To address these questions, we used hamster (chronic model) and hACE2.Tg mice (acute model) to evaluate the protective efficacy of WS, TC, and PL and the immunological correlates of protection in COVID-19. Our data from the hamster challenge study showed that prophylactic dosing of WS, but not TC or TC in combination with PL (TC+PL), was able to significantly reduce body weight loss. In line with this, WS dosing showed significantly decreased lung viral load, pulmonary pathology, and suppression of inflammatory cytokine mRNA expression. In contrast, the TC group showed robust anti-inflammatory potential but no viral load and pathology alleviation. Next, we used cellular T-cell assay to show potent inhibition of Th1, Th2, and Th17 differentiation in presence of WS, while TC was found to inhibit Th1, Th2, and Th17 differentiation only at higher doses. To understand the effector immune population, we used hACE2.Tg model and show that WS administration results in boosting the immunosuppressive environment in SARS-CoV-2 infected mice which leads to amelioration of pulmonary pathology and significant protection against COVID-19 morbidity and mortality. Together, we provide data from moderate and acute SARS-CoV-2 animal challenge studies suggesting robust protective efficacy by prophylactic WS and determining the immune correlates of protection. Our *in-vitro* and *in-vivo* results support the translational value of WS against COVID-19 and provide the basis for further clinical evaluations.

## Materials and methods

WS, TC, and PL extract was provided by National Medicinal Plant Board and was used as per pharmacopeial standards in the current study for both *in-vitro* and *in-vivo* evaluations.

### Animal ethics and biosafety statement

6-8 weeks of K18-humanized ACE2 transgenic mice (hACE2. Tg mice) were initially procured from Jackson’s laboratory and then bred and maintained at the small animal facility (SAF), THST. Golden Syrian hamsters (6-9 weeks) were procured from the Central drug research institute (CDRI) and were used for experimentation post-quarantine. The animals were randomly divided into 5 groups based on their body weight viz uninfected (UI), Infected (I), Infected treated with remdesivir (I+RDV), Infected treated with WS (I+WS), Infected treated with TC (I+TC) and infected treated with TC+PL (I+TC+PL). The hamster prophylactic WS, TC or TC+PL group started receiving twice-daily oral doses of 130 mg/kg (0.5% CMC preparation) 5 days before the challenge and continued till the endpoint. The hACE2 transgenic mice group started receiving prophylactic WS, TC or TC+PL group started receiving twice-daily oral doses of 78 mg/kg (0.5% CMC preparation) 5 days before the challenge and continued till the endpoint. The hamster remdesivir control group received 15mpk (subcutaneous: sc) on 1 day before and 1 day after the challenge while hACE2 transgenic mice received 25mpk (intraperitoneal: ip) injections of remdesivir started on the same day of infection and continued till the end point. The animals were shifted to ABSL3 1 day prior to the challenge. Live intranasal infection of SARS-CoV-2 SARS-Related Coronavirus 2, Isolate USA-WA1/2020) 10^5^PFU/100μl (for hamster) and 10^5^PFU/50μl (for mice) or with DMEM mock control was established with the help of catheter under mild anesthetized by using ketamine (150mg/kg) and xylazine (10mg/kg) intraperitoneal injection inside ABSL3 facility (29–34). Experimental protocols related to handling and experimentation was approved by RCGM, institutional biosafety, and IAEC (IAEC/THSTI/105) animal ethics committee.

### Preparation and characterization of WS, TC, PL extract

1g of dry powder of WS (roots), TC (stem), and PL (seeds) were dissolved in 100 ml of water at 37°C overnight in a shaker incubator to obtain the herbal extracts. The following day, the suspended extract was centrifuged at high speed 10000 x g for 30 min. The supernatant thus obtained was filtrated by using a 0.45 filter. The filtrate obtained was assumed to be 100% aqueous extract which was further diluted in water to achieve a dosing concentration. The filtrate was further used for evaluating the composition and was previously published (28).

### Virus culture and titration

Dulbecco’s Modified Eagle Medium (DMEM) complete media containing 4.5 g/L D-glucose, 100,000 U/L Penicillin-Streptomycin, 100 mg/L sodium pyruvate, 25mM HEPES and 2% FBS was used to propagate and titrate SARS-Related Coronavirus 2, Isolate USA-WA1/2020 virus in Vero E6 cell line. The plaque-purified stocks of virus were prepared and used inside at ABSL3 facility at IDRF, THSTI in accordance with the IBSC and RCGM protocols.

### Gross clinical parameters of SARS-CoV2 infection

For mice experiment the endpoint of the study was day 6 post-challenge, while for hamster study the endpoint was 4 days post-challenge. The animal body weight was recorded for everyday post challenge. At the end point all the animals were sacrificed and a necropsy was performed to investigate lungs and spleen. Gross morphological changes were recorded and imaging was performed for excised lungs and spleen. For histological analysis, left lower lobe of the lung was excised and fixed in 10% formalin (31, 33, 35). Lungs were homogenized in Trizol for RNA isolation while spleen was either homogenized (hamster) or used for flow cytometry (hACE2 transgenic mice) (30). The homogenized samples were immediately stored at -80 °C till further use. Serum samples isolated from blood w immediately stored at -80 °C till further use.

### Viral load

Isolated lung was homogenized in 2ml Trizol reagent (Invitrogen) and RNA was isolated by Trizol-Choloform method. Yield of RNA was quantitated by nano-drop and 1 µg of RNA was use to reverse-transcribed to cDNA using the iScript cDNA synthesis kit (BIORAD; #1708891) (Roche). 1:5 diluted cDNAs was used for qPCR by using KAPA SYBR^®^ FAST qPCR Master Mix (5X) Universal Kit (KK4600) on Fast 7500 Dx real-time PCR system (Applied Biosystems) and the results were analyzed with SDS2.1 software (30, 33). Briefly, 200 ng of RNA was used as a template for reverse transcription-polymerase chain reaction (RT-PCR). The CDC-approved commercial kit was used for of SARS-CoV-2 N gene: 5′-GACCCCAAAATCAGCGAAAT-3′ (Forward), 5′-TCTGGTTACTGCCAGTTGAATCTG-3′ (Reverse). Hypoxanthine-guanine phosphoribosyl transferase (HGPRT) gene was used as an endogenous control for normalization through quantitative RT-PCR. The relative expression of each gene was expressed as fold change and was calculated by subtracting the cycling threshold (Ct) value of hypoxanthine-guanine phosphoribosyl transferase (HGPRT-endogenous control gene) from the Ct value of the target gene (ΔCT). Fold change was then calculated according to the formula POWER(2,-ΔCT)*10,000 (36, 37).

### qPCR from splenocytes

RNA isolated from spleen samples were converted into cDNA as described above. Thereafter, the relative expression of each gene was expressed as fold change and was calculated by subtracting the cycling threshold (Ct) value of hypoxanthine-guanine phosphoribosyl transferase (HGPRT-endogenous control gene) from the Ct value of target gene (ΔCT). Fold change was then calculated according to the formula POWER (2, -ΔCT)*10,000 (36–38). The list of the primers is provided in **Table 1** as follows.

**Table 1:** Mouse qPCR primers.

Gene	Forward	Reverse
HGPRT	GATAGATCCACTCCCATAACTG	TACCTTCAACAATCAAGACATTC
tryptase β2	TCGCCACTGTATCCCCTGAA	CTAGGCACCCTTGACTTTGC
chymase	ATGAACCACCCTCGGACACT	AGAAGGGGGCTTTGCATTCC
muc1	CGGAAGAACTATGGGCAGCT	GCCACTACTGGGTTGGTGTAAG
Sftp-D	TGAGCATGACAGACGTGGAC	GGCTTAGAACTCGCAGACGA
Eotaxin	ATGTGCTCTCAGGTCATCGC	TCCTCAGTTGTCCCCATCCT
PAI-1	CCGTGGAACCAGAACGAGAT	ACCAGAATGAGGCGTGTCAG
IFNγ	TGTTGCTCTGCCTCACTCAGG	AAGACGAGGTCCCCTCCATTC
TNFα	AGAATCCGGGCAGGTCTACT	TATCCCGGCAGCTTGTGTTT
IL13	AAATGGCGGGTTCTGTGC	AATATCCTCTGGGTCTTGTAGATGG
IL17A	ATGTCCAAACACTGAGGCCAA	GCGAAGTGGATCTGTTGAGGT
IL10	GGTTGCCAAACCTTATCAGAA ATG	TTCACCTGTTCCACAGCCTTG
IL6	GGACAATGACTATGTGTTGTTAGAA	AGGCAAATTTCCCAATTGTATCCAG

### Histology

Formalin-fixed samples of lungs were embedded in paraffin blocks, sectioned and stained with hematoxylin and eosin dye as previously described (35, 36). Strained lung samples were then analysed and imaged at 40X. Histological assessment for pathological features was done by professional histologist in a blinded manner and scoring was carried out on a scale of 0-5 (where 0 indicated the absence of histological feature while 5 indicated the highest score). Disease index score was calculated by the addition of all the individual histological scores.

### *In vitro* differentiation of T cells

The single cell suspension was prepared from spleen and lymph nodes of 6–8 weeks old C57BL/6 mice. The cells were activated using soluble anti-CD3 (2ug/ml) and differentiated into Th1 conditions by adding recombinant mouse IL-12 (15ng/ml) cytokine or Th2 conditions by adding recombinant mouse IL-4 (15ng/ml) cytokine or Th17 conditions by adding TGF-beta (2ng/ml) plus IL-6 cytokine (25ng/ml) (37, 39). WS or TC was added in concentrations ranging from 10ug/ml to 1000ug/ml at the start of culture. Cells were harvested after 72 hours of culture. Intracellular cytokine staining was performed to check the expression of IFN-gamma, IL-4 and IL-17 cytokine for Th1, Th2 and Th17 cells respectively.

### Intracellular cytokine staining

Surface markers were stained for 15–20 min in room temperature in PBS with 1% FBS, then were fixed in Cytofix and permeabilized with Perm/Wash Buffer using Fixation Permeabilization solution kit and stained anti-IL-17A; anti-IFN-gamma, anti-IL-4 diluted in Perm/Wash buffer. All antibodies were used in 1:500 dilution. The cells were then taken for flow cytometry using BD FACS-CantoII and data was analyzed with FlowJo software (32, 36, 37).

### Isolation of murine BMDNs and human peripheral neutrophils

Murine BMDNs were isolated according to the method described by Rizvi et al., 2022 from long bones of C57BL/6 wild-type male mice (12–16 weeks, 20–25 g) (31). After flushing the long bones with HBSS + 0.1% BSA, BMDNs were collected between the 81% and 62% layers of the Percoll (Sigma GE17-0891-02) density gradient. Cell viability and purity were checked by Trypan blue and anti-Ly6G (Thermo 14-5931-82) and anti-CD11b (Thermo 14-0112-82) antibodies, respectively. Similarly, human PMNs were also isolated from the peripheral blood of healthy individuals, after sedimenting the RBCs with 6% dextran at 37°C. Isolated neutrophils were assessed by CD15 (Thermo 14-0159-82) labeling for their purity. All the studies on mice were approved by the institutional animal (THSTI/105) and human (THS1.8.1/100) ethical committees, DBT-THSTI, Faridabad.

### Cell viability assay

Different concentrations of the extracts, ranging from 100-1000 µg/ml were used to determine, if any, cytotoxicity on neutrophils up to 240 min (40). 1.0x10^6^ per ml cells were incubated with propidium iodide (PI, 50 μg/ml, Sigma P4170) for 15 min and analyzed on BD FACS Canto cell analyzer (BD Biosciences, USA).

### Intracellular ROS and mtROS analysis

Both cytosolic and mitochondrial ROS were measured with DCFH-DA (10 μM, Sigma D6883) and MitoSOX (10 μM, Thermo M36008), respectively as previously reported (40). Extract pre-incubated cells (1.0 x 10^6^ cells/ml) were treated with different interventions such as TRLM (10 μM, MedChem HY-109104), PMA (10-100 nM, Sigma P1585), A23187 (1-5 μM, Sigma C5149), ionomycin (1-4 μM, Sigma I9657), NAC (10 μM, Sigma A9165), and MitoTEMPO (10 μM, Sigma SML0737). DMSO (0.1%) was used as a control. A minimum of 10,000 events were acquired for each sample using BD FACS Canto II.

### NETosis assay

5.0 x 10^4^ cells were incubated with both the extract for 60 min at 37°C, followed by treatment with TRLM (10 uM), PMA (10 uM), A23187 (1 uM), ionomycin (1 uM), or vehicle (DMSO 0.1%). SYTOX Green (100 nM, Thermo S7020) was used to monitor the fluorescence at different time periods up to 240 min in a plate reader at 37°C (Synergy 2; BioTek) as described earlier (Rizvi et al., 2022). Additionally, immunofluorescence images were also developed using anti-MPO (Santa Cruz Sc390109) and anti-H4Cit3 (Sigma 07-596) antibodies by confocal microscope (Olympus FV3000) at 100X resolution.

### Phagocytosis and bactericidal assay

Phagocytosis was accessed by adding PE-labelled latex beads (Sigma L2778) to extract pre-treated PMNs (1.0 x 10^4^) at a 1:50 ratio using FACS (41). The bactericidal activity of neutrophils was accessed by first incubating the cells with extracts and then treating them with kanamycin-resistant *E. coli* for 30 min at 37°C. Internalized bacteria were plated on LB agar after the lysis of PMNs. The killing activity is expressed as a percent of CFU in the presence/absence of PMNs.

### Statistical analysis

All the experiments have been carried out independently in triplicate. Results are being expressed as mean ± SEM. Multiple group comparisons have been performed using one-way ANOVA followed by the Bonferroni test using GraphPad Prism 8. The differences have been considered as statistically significant when the *p*-value was < 0.05.

## Results

### Prophylactic use of WS, but not TC, limits SARS-CoV-2-induced pulmonary pathology in hamsters

**Figure 1:**
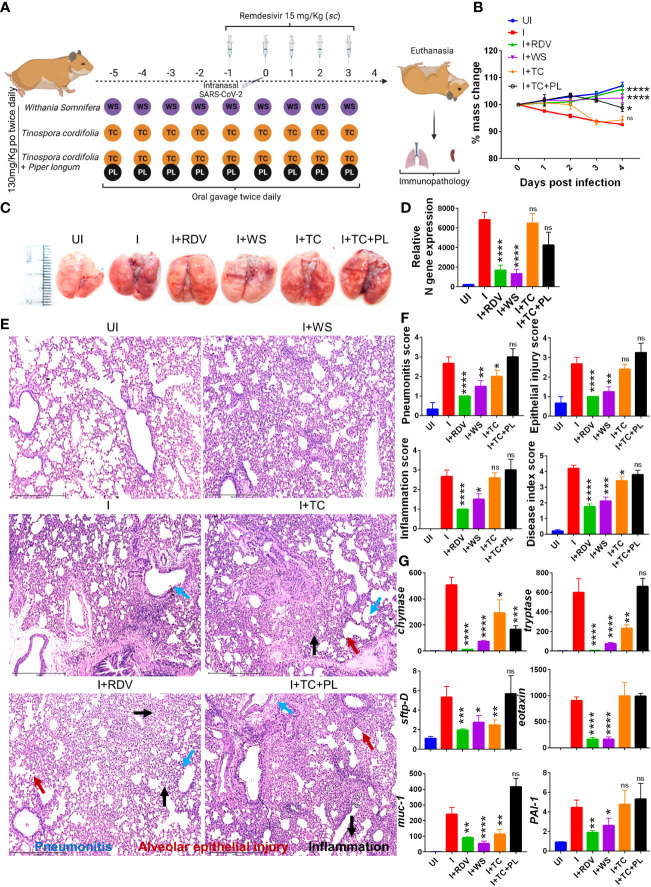
Prophylactic efficacy of selected herbal extract on the SARS-CoV-2 infected hamsters. **(A)** Schematic representation of dosing regimen for prophylactic treatment of WS, TC or TC in combination with PL, positive control remdesivir (RDV), infection control (I) or uninfected hamster group. All the animals except the uninfected control was intranasally challenged with 10^5^ pfu SARS-CoV-2 on day 0 and sacrificed on 4-day post infection (dpi). **(B)** Body mass of the animals were monitored post challenge and was plotted as %age change as compared to its day 0 body mass. **(C)** Representative images of harvested lungs post necropsy. **(D)** Relative viral load by N gene expression by qPCR shown as bar graph mean ± SEM. Histological analysis of left lung lower lobe was carried out post necropsy. The samples were fixed in 10% neutral formalin solution, paraffin embedded, sectioned and hematoxylin (H) & eosin **(E)** stained. Stained sections were then imaged at 10X and assessed by trained pathologist for histological features. **(E)** Representative images of HE stained lungs showing pneumonitis (blue), bronchitis (red), epithelial injury (green) and inflammation (yellow). **(F)** Blinded pathological score for pneumonitis, bronchitis, lung injury, epithelial injury and inflammation as assessed by trained pathologist. **(G)** mRNA expression of key genes involved in cellular injury of lungs. For each experiment N=5. One way-Anova using non-parametric Kruskal-Wallis test for multiple comparison. *P < 0.05, **P < 0.01, ***P < 0.001, ****P < 0.0001.

To determine and evaluate the therapeutic potential of WS and TC (two commonly used traditional herbs) against COVID-19, we used a previously established hamster model for SARS-CoV-2 infection which has been shown to mimic moderate COVID-19 pathology (30, 31, 33, 42, 43). The dosing regimen involved prophylactic intra-gastric administration of WS, TC, or TC+PL for 5 days before intranasal SARS-CoV-2 challenge in hamsters which was continued till the end point of the study (4 days post-infection, dpi). RDV was used as a prototypic anti-viral as a positive control to compare the *in vivo* results. Though, RDV in clinical trials results in marginal protection against morbidity, in animal studies RDV has been shown to provide robust protection against COVID-19. The schematic summary of the dosing regimen and study design is shown in **Figure 1A**. Our hamster challenge data indicated that prophylactic dosing of WS was able to significantly reduce the body mass loss following SARS-CoV-2 infection as compared to the (I+WS). This protection against mass loss was found to be similar to that of the RDV-treated group (**Figure 1B**). However, both TC and TC+PL treated groups showed 4-8% body mass loss at 4 dpi when compared to the uninfected group (UI) (**Figure 1B**). Since protection in body mass loss is correlated with the decreased lung viral load and pathology, we next examined the gross morphological manifestation in excised lungs post necropsy and the corresponding lung viral load. Our data show significantly reduced pathological features and relative N gene expression in the lungs in the WS treatment group, as compared to the infected group, which was similar to the reduction seen in the RDV group. The TC and TC+PL group showed no significant reduction in the pathology and viral load in the lungs (**Figures 1C, D**).

In a significant percentage of clinical cases, COVID-19 is characterized by inflammation in the lungs leading to pneumonitis and cellular injury (44). We, therefore, set out to understand the degree of protection in pulmonary pathology by WS, TC, and TC+PL groups. Blinded-random histopathological assessment of the lung sections by a trained pathologist showed robust overall protection in the hamsters receiving WS in terms of overall mitigation in pneumonitis, bronchitis, epithelial injury, lung injury, and inflammation score which was Together, we provide pre-clinical data from mild and severe infection models suggesting robust protection by WS against COVID-19 through its broader immunomodulatory activity. Our study supports the evaluation of WS alone or as a formulation for therapeutic intervention against acute viral infections.

Similar to the degree of protection in the RDV-treated group. TC and TC+PL groups, however, failed to alleviate the overall pathological score of the lungs (**Figures 1E, F**). Next, to understand the mechanism we evaluated the expression of genes involved in lung injury. Chymase and tryptase are effector enzymes secreted by mast cells that have been implicated in COVID-19 pulmonary pathology (45, 46). On the other hand, secretion of mucin-1 (muc1) and surfactant protein D (sftp-D) are important defense mechanisms in the lungs against pathogenic infection, while elevated plasminogen activator inhibitor-I (PAI-1) is a risk factor for thrombosis and has been shown to be correlated with COVID-19 severity (30). Eotaxin is a lung injury-associated gene whose lung expression is upregulated in the case of severe COVID-19 (30, 47). Our data showed that both WS and TC were able to significantly decrease the mRNA expression of chymase, tryptase, sftpD, and muc1, though the fold change inhibition observed in the WS group was dramatically more prominent than that observed in the TC group. Notably, WS, but not TC, group showed a decrease in exotoxin and PA1 as well (**Figure 1G**). However, the combinatorial effect of TC with PL showed no alleviation in the gene expression markers for lung injury. In conclusion, we show that hamsters receiving prophylactic dosing of WS but not TC or TC in combination with PL alleviates the pulmonary pathology induced by COVID-19.

### Prophylactic WS promotes the anti-inflammatory response to COVID-19

**Figure 2:**
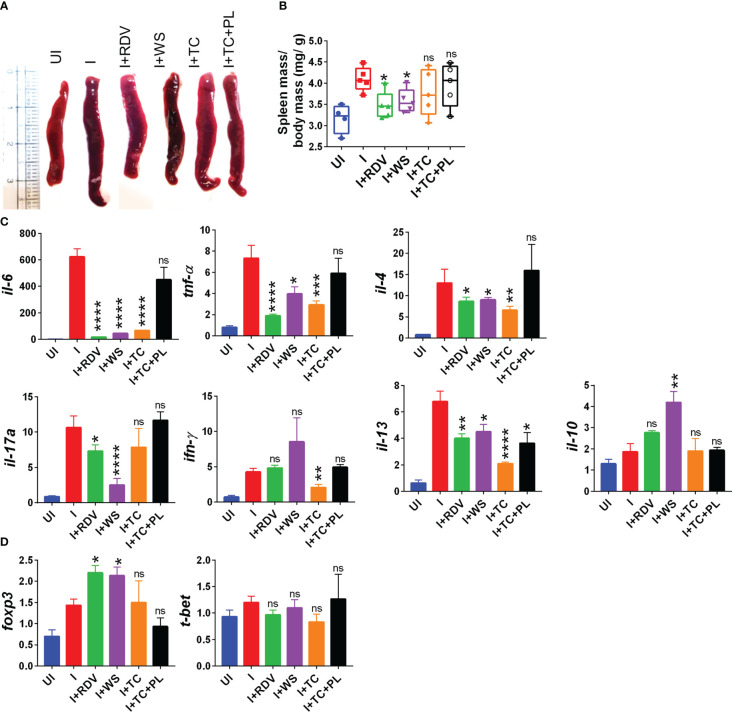
Immunomodulatory effects of WS or TC on the infected hamsters. Immunomodulatory activity of prophylactic treatment of WS or TC on infected vs uninfected hamsters were studied. **(A)** representative spleen images harvested post necropsy. **(B)** changes in spleen mass to body mass ratio for different groups **(C)** modulation in the mRNA expression of pro-inflammatory cytokines and **(D)** transcription factors. For each experiment N=5. One way-Anova using non-parametric Kruskal-Wallis test for multiple comparisons. *P < 0.05, **P < 0.01, ***P < 0.001, ****P < 0.0001.

During the active phase of SARS-CoV-2 infection, immune cells are recruited in the lungs leading to an aggressive inflammatory response that is correlated to morbidity and mortality (44). Previous studies have shown that the inflammatory profile of the lungs corroborates well with the inflammatory profile of the spleen in COVID-19 animal models (30, 31). Furthermore, splenomegaly has been shown to be one of the crucial COVID-19 severity indicators in the hamster model (30). In line with the previously published reports, we found a significant increase in the spleen length and mass in the infected hamsters while the hamsters receiving WS and RDV showed a significant reduction in the body spleen length and mass increase as compared to the infected control (**Figures 2A, B**). Severe COVID-19 patients have elevated pro-inflammatory cytokines and diminished anti-inflammatory cytokines levels. Therapeutic drugs such as DXM which were successful in decreasing the pro-inflammatory cytokines were found to be effective in clinical trials. Therefore, we tested the immune-modulatory potential of WS, TC, and TC+PL in SARS-CoV-2-infected hamsters. Our mRNA expression data from the spleen shows that both WS and TC showed potent anti-inflammatory potential in lowering the expression of *IL-6, IL4, IL13*, and *TNF-α*. Notably, WS showed inhibitory potential for *IL*-17 cytokine which is pathogenic for pulmonary injury, and significantly boosted the expression of *IL-10* cytokine and *foxp3* transcription factor which are crucial for the induction of regulatory T cells (Tregs) (**Figures 2C, D**). There were no significant changes observed in the expression of *IFN-γ* cytokine and *t-bet* transcription factor which is responsible for the induction of Th1 response. The TC+PL group also showed non-significant modulations compared to the I group. Together, we found both WS and TC to show immunomodulatory potential in COVID-19 hamsters. WS was more potent in the induction of anti-inflammatory response.

### Effect on TRLM-PMA/ionophore-stimulated ROS and mtROS production in human PMNs and murine BMDNs

Neutrophils engage the pathogens by TLRs *via* recognizing PAMPs: among the discovered TLRs, endosomal TLR 7/8 binds viral single-stranded RNA as in SARS-CoV-2. Various TLR7/8 agonists induce neutrophil activation (48) however, little is known about a putative link between TLR7/8 signaling and neutrophil responses. In the present study, we found a significant increase in different neutrophil functions against priming of TLR7/8 by TRLM prior induction with PMA and/or ionophores.

**Figure 3:**
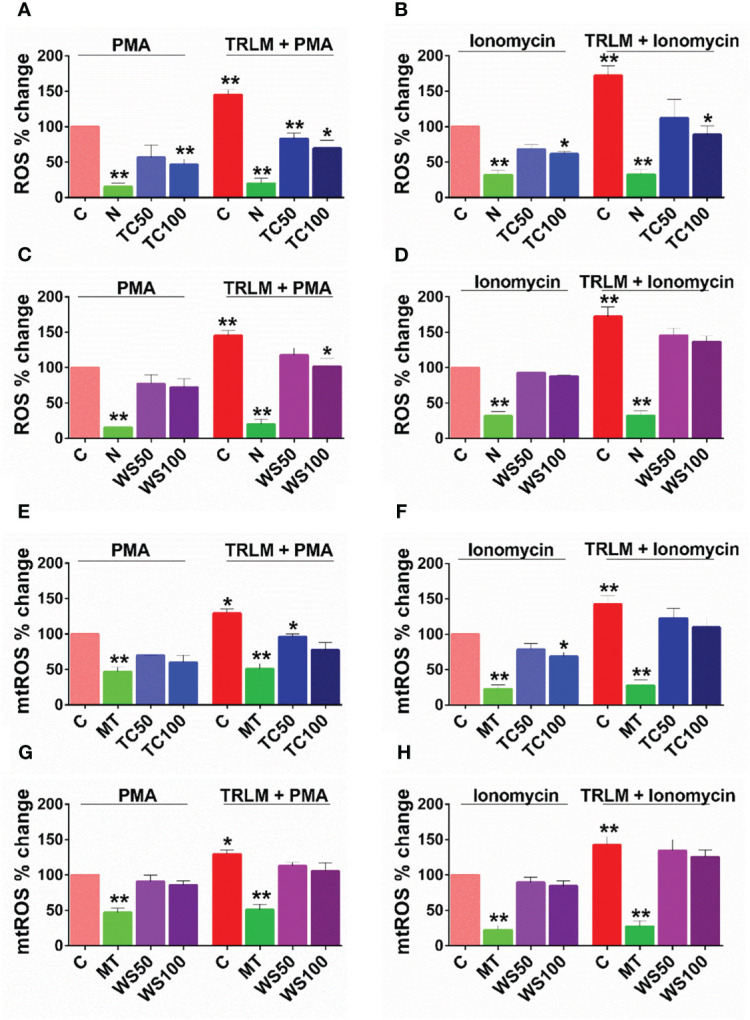
Effect of WS and TC on TRLM primed-PMA/ionomycin induced NETs formation in human PMNs. After pre-incubation with different concentrations TC and WS, PMNs were treated with TRLM (10 µg/ml) for 30 min and stimulated with sub-maximal concentration of PMA (12.5 nM) and ionomycin (2 µM) for 30 min. SYTOX Green (100 nM) was used to monitor extracellular DNA release using a plate reader (**A, B**: TC; **C, D**: WS). Total MFI in each experimental condition is expressed as Mean ± SEM of min 3 experiments. NETosis in human PMNs was also monitored using immunofluorescence imaging with DAPI (blue), anti-MPO antibody (green), and anti-H4Cit3 antibody (red, **E–H**). Representative fields are shown at 100X with a scale bar of 10 µm. Bar diagram represents quantification of percent NETs forming cells as calculated from five transects from three independent experiments. Statistical analysis consisted of one-way ANOVA followed by Bonferroni’s test (*p < 0.05, **p < 0.01, vs respective control groups; C, control; V, VAS2870; D, Diltiazem; WS100, WS 100 μg/ml; WS300, WS 300 μg/ml; TC100, TC 100 μg/ml; TC300, TC 300 μg/ml.

*WS* and *TC* have been extensively characterized elsewhere as an alternative or complementary remedy for oxidative and inflammatory diseases owing to the presence of a range of alkaloids, polyphenols, terpenes, flavonoids, coumarins and other phytochemicals. Therefore, to determine the effect of WS and TC on ROS and mtROS production, DCF-DA and mitoTEMPO were added respectively, to the cells followed by priming with TRLM and induction with PMA and ionophores. A marked decrease in ROS production in TC-treated PMNs, from 43% (50 μg/ml) to 52% (100 μg/ml, p<0.05) was observed when stimulated with TRLM-PMA whereas TRLM-ionomycin led to a decrease of 35% at 50 μg/ml and 49% at 100 μg/ml, p<0.05 (**Figures 3A, B**). WS also had similar effects in limiting the formation of superoxide anions elicited by TRLM-PMA (19% at 50 μg/ml and 30% at 100 μg/ml, p<0.05) or TRLM-ionomycin (maximum decrease of 21% at 100 μg/ml, p<0.05) (**Figures 3C, D**). A similar trend was also seen in murine datasets (**Figures S1A–D**). Further, the ability of TC, 100 μg/ml to inhibit mtROS production in PMNs revealed a 40% and 23% reduction with TRLM-PMA and TRLM-ionomycin respectively (**Figures 3E–F**). A somewhat lower percent reduction was seen in WS- exposed cells with TRLM-PMA (19%), and TRLM-ionomycin (12%) at 100 μg/ml of WS (**Figures 3G, H**). Similar to the TC and WS effect on PMNs, murine cells also showed more potent inhibition on TRLM-PMA mediated cytosolic and mitochondrial radical production (**Figures S1E–H**). Notably, WS and TC both were comparatively more efficient in reducing PMA-mediated ROS and mtROS production in neutrophils from humans or mice.

### Effect on NETosis in human PMNs and murine BMDNs

Since sera and postmortem lung biopsies from COVID-19 patients have a high concentration of NET components especially in the inflammatory interstitial lesions and airways (49), elucidating their detailed mechanism could be highly useful. Although NETs formation has been considered a defensive microbicidal phenomenon to exterminate the invading foreign pathogens (50) but a loss of its control and their persistent presence in inflammation results in host tissue damage.

**Figure 4:**
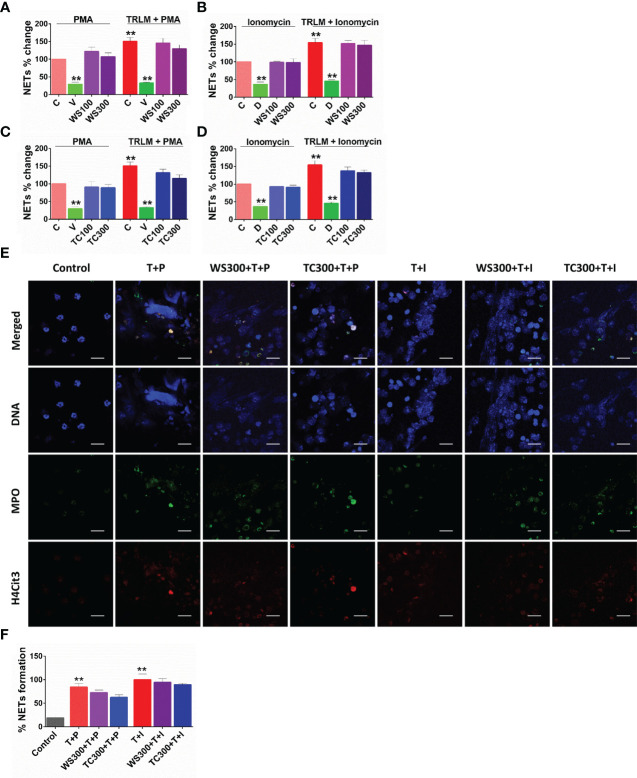
Effect of WS and TC on TRLM primed-PMA/ionomycin induced cytosolic ROS and mtROS production in human PMNs. PMNs pre-incubated at different concentrations of TC and WS were treated with TRLM (10 µg/ml) for 30 min and stimulated with sub-maximal concentration of PMA (12.5 nM) and ionomycin (2 µM) for 30 min. DCF-DA (10 µM) and MitoSOX (10 µM) were used for cytosolic ROS and mtROS detection, respectively using flow cytometry. All the data are represented as Mean ± SEM, n = min 3 per group, and statistical analysis consisted of one-way ANOVA followed by Bonferroni’s test **(A–F)** (**p < 0.01, vs respective control groups;. C, control; N, N-acetyl cysteine; MT, MitoTEMPO; WS50, WS 50 μg/ml; WS100, WS 100 μg/ml; TC50, TC 50 μg/ml; TC100, TC 100 μg/ml.

A steep rise in NETosis of more than 50% was observed in PMNs primed with TRLM before exposing PMA or ionophores. Supplementing the cells with TC but not WS could inhibit double-stranded DNA release, a hallmark for NETs formation. TC exhibited an inhibitory effect on NETs in a concentration-dependent manner. A low concentration of TC has no significant effect on the inhibition of NETs in neutrophils, however, higher concentrations exerted an inhibitory effect on the release of dsDNA. PMA-induced NETosis in human PMNs was reduced from 13% to 25% after treatment with 100 and 300 µg/ml, p<0.05 of TC respectively (**Figure 4A**). In contrast, with TRLM-ionomycin stimulation TC did not exert a noticeable reduction in DNA release; a maximum of 15% inhibition was seen at 300 μg/ml (**Figure 4B**). Treatment of cells with WS did not reveal a significant down-regulation of NETosis (**Figures 4C, D**). Further, similar effects were replicated in the mouse model also using murine BMDNs as shown in **Figures S1I–L**. Our immunofluorescence images further corroborated the fluorimetry data. **Figures 4E, F** showed that the pre-treatment of PMNs with 300 μg/ml TC prevented the diffused and web-like state of TRLM-PMA treated cells as evidenced by the reduction of percent NETs forming cells, MPO, and H4Cit3 expression. Incubation of TC with the TRLM-ionomycin group did not result in much elimination of characteristic DNA fibers extrusion, except with shrinkage of nuclear diameter. Results obtained thus indicate that TC might contribute to the regulation of neutrophil NETs formation *via* modulating the PMA-mediated signaling pathways involved in NETosis.

### Effect of WS on phagocytosis by human PMNs

In addition to degranulation and NETs, phagocytosis is another critical anti-microbial function of neutrophils. The measurable effect of WS and TC on neutrophils led us to study the role of these extracts on the phagocytic potential of these immune first responders. TC and WS showed a modest reduction of 17% and 13% (p<0.05), respectively only at the high concentration (300 µg/ml, **Figures S2A, B**), while 1-100 µg/ml of both the herbal extracts did not elicit any noticeable change. Also, pre-treatment of human peripheral neutrophils with TC and WS did not impart any significant effect on the killing activities of phagocytes; approximately 74% and 41% reduction in *E. coli* growth was observed when bacteria were incubated with TC and WS-treated PMNs, respectively (**Figures S2C, D**).

### Effect of WS, and TC on Th1, Th2, and Th17 polarization

**Figure 5:**
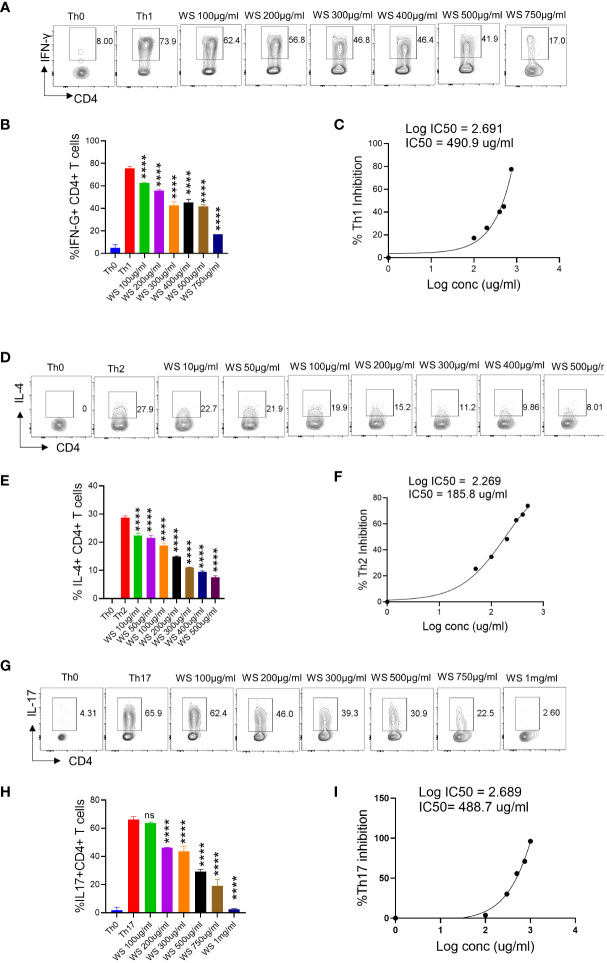
Dose kinetics of WS response on *in vitro* differentiation of Th1, Th2 and Th17 cells from naïve CD4+ T cells. Sorted naïve CD4+ T cells from mouse spleen and lymph nodes were activated using soluble anti-CD3 antibody and differentiated into helper T (Th)2 **(A, B)**, Th17 cells **(D, E)** and Th1 subtypes **(G, H)** by using different cytokines viz recombinant mouse IL-4; TGF-β + IL-6 and IL-12 cytokines respectively. WS was added in concentrations ranging from 10ug/ml to 1000ug/ml initially at the time of cell seeding. After 72 h of incubation IL-4, Il-17 and IFN-gamma production was measured respectively for Th2, Th17 and Th1 cells by intracellular cytokine staining. IC50 values were calculated using Graph pad prism software **(C, F, I)**. ****P < 0.0001 by one-way ANOVA.

T helper cell subset responses determine the clinical outcome of SARS-CoV-2 infection (51, 52). An appropriate Th1 cell response is required to clear the virus when the infection is initially established. However, prolonged Th1 cell activation precedes cytokine storm and priming of Th2 responses, leading to a poor prognosis (51, 53). Patients with severe Covid infection show high levels of IL-17 and GM-CSF (44, 54). Th17 cells lead to the recruitment of neutrophils and increase vascular permeability and leakage, causing lung damage (55). Thus, preventing the hyperactivation of pro-inflammatory T cells (Th1, Th2, and Th17) will help reduce the disease’s severity. To study the immunomodulatory role of WS, we studied the effect on *in vitro* differentiation of different Th cell subsets like Th1, Th2, and Th17 cells (**Figure 1**). WS showed effective inhibition in the differentiation of Th1, Th2, and Th17 cells with an increase in doses (**Figures 5A–I**). IC50 values were calculated to compare its efficacy in inhibiting different Th cell subsets. The IC50 value of WS for Th1, Th2, and Th17 cells inhibition were 490.9ug/ml, 185.8 ug/ml, and 488.7ug/ml respectively (**Figures 5C, F, I**). These observations show that WS is a more potent inhibitor of Th2 cells, followed by Th17 and Th1 cells. We also studied the effect of TC on *in vitro* differentiation of helper T cell subsets Th1, Th2, and Th17 cells (**Supplementary Figures S3A–I**). It showed a marginal inhibition of Th1, Th2, and Th17 cells with an increase in doses. The IC50 value of TC for Th1, Th2, and Th17 cells inhibition was 1294 ug/ml, 1330 ug/ml, and 1679 ug/ml (**Supplementary Figures S3A–I**). These observations show that TC is not as good an inhibitor of pro-inflammatory T-cell differentiation as compared to WS. DXM was used as a positive control since it is a well-known immunosuppressive drug (**Supplementary Figures S4A–I**). IC50 values of DXM were 517.6nM, 1364pM, and 3162pM for Th1, Th2, and Th17 cells respectively (**Supplementary Figures S4A–I**). Together, through our *in-vitro* assay, we show that WS exhibits immune-suppressive potential and could inhibit the differentiation of Th1, Th2, and Th17 cells similar to the DXM-mediated inhibition.

### WS mitigates COVID-19 pathology and improves overall survival in hACE2.Tg mice

**Figure 6:**
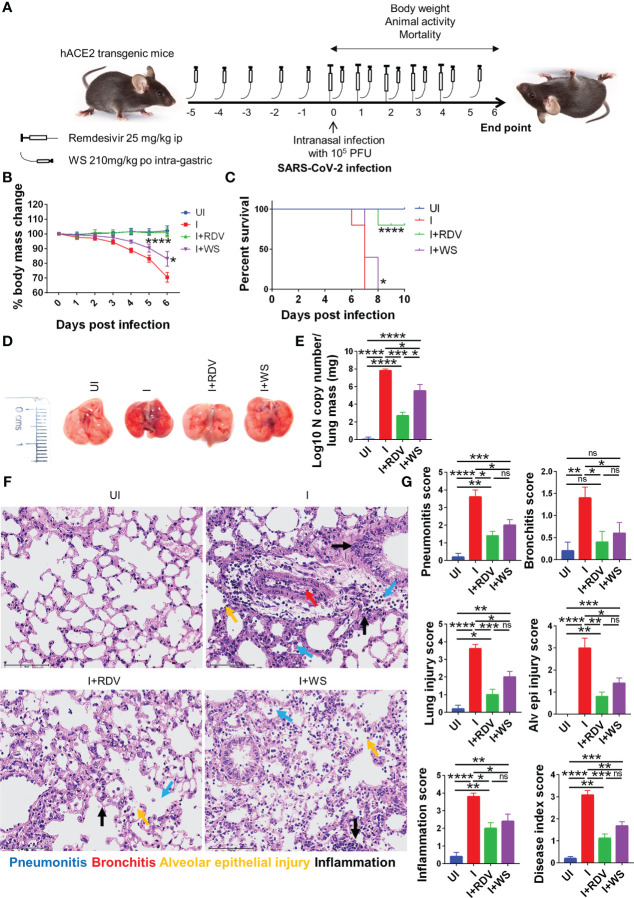
Assessment of protective efficacy of WS in acute SARS-CoV-2 infection model of hACE2 transgenic mice. To evaluate the effect of prophylactic treatment of WS on severe SARS-CoV-2 infection, we used hACE2 mice model for acute infection and compared it with RDV control. **(A)** Schematic representation showing treatment regimen for WS and RDV. Mice were intranasally infected with SARS-CoV-2 and **(B)** %age changes in body mass and **(C)** mortality was monitored and plotted. **(D)** Representative excised lung images 6 days post infection **(E)** Lung viral load presented as Log10 N copy number **(F)** Lower lung lobe was used for HE staining **(G)** and assessed for pathological features by blinded scoring by trained pathologist. For each experiment N=5. One way-Anova using non-parametric Kruskal-Wallis test for multiple comparisons. *P < 0.05, **P < 0.01, ***P < 0.001, ****P < 0.0001.

Screening of anti-viral or immunomodulatory drugs against COVID-19 has so far relied on two *in vivo* models viz hamster and hACE2.Tg mice model both of which mimic clinical symptoms of COVID-19 yet significantly differ in the disease pathology (30, 31, 43, 56). While hamsters have been shown to develop mild to moderate COVID-19 pathology following intranasal infection and mimic the majority of the clinical cases, hACE2.Tg mice, on the other hand, are a lethal model for SARS-CoV-2 infection and result in severe respiratory distress leading to 100% mortality by 6-8 days post-infection (dpi). In order to understand the protective efficacy of WS during acute infection, which is known to occur in a less but a significant number of clinical cases, we used hACE2.Tg mice model (**Figure 6A**). TC and TC+PL were not evaluated in hACE2.Tg mice, since they did not show significant protection in the hamster model previously. Our data from hACE2.Tg mice challenge study showed about 8-10% recovery in the body mass in the WS-treated group as compared to the I control (**Figure 6B**). The overall survival of the hACE2.Tg mice improved by 2 days in WS treated group which was marginal yet significant as compared to the I control (**Figure 6C**). We next examined the gross morphological changes, viral load and pathological features in the excised lungs post necropsy on 6 dpi. Our data show that the WS group showed significant alleviation in gross morphological changes and lung viral load in the WS group as compared to the I control (**Figures 6D, E**). The H & E histopathological assessment results showed robust protection in the overall pathological scores in the WS group as compared to the I control (**Figures 6F, G**). RDV group showed log10 2-fold decreased lung viral load, however, the pathological disease index score of RDV and WS was found to be similar. Taken together, we found significant mitigation in COVID-19 pathology and lung viral load in the WS group which is reminiscent of robust protective efficacy.

### WS treatment results in the boosting of MDSCs in hACE2.Tg mice

**Figure 7:**
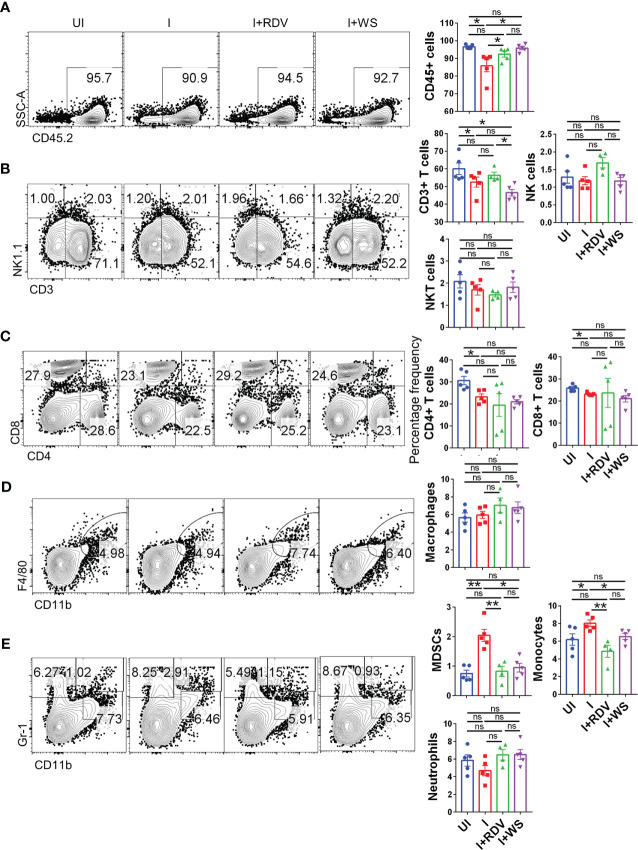
Changes in the major immune populations of infected hACE2 mice with or without treatment. Flow cytometry-based quantitation was done to evaluate changes in the major immune population in the lymph nodes of sacrificed animals at 6 dpi. The % age frequency was plotted as bar graph along with the representative contour plot **(A)** CD45+ population **(B)** CD3+ T lymphocytes, NK cells and NKT cells **(C)** CD4+ T helper cells and CD8+ T cytotoxic cells **(D)** Macrophages **(E)** Monocytes, neutrophils and MDSCs population. For each experiment N=5. One way-Anova using non-parametric Kruskal-Wallis test for multiple comparisons. *P < 0.05, **P < 0.01.

COVID-19 is characterized by lymphopenia resulting in dysregulation of immune profile and function (57). To test the immunomodulatory potential of WS in mice infected with SARS-CoV-2, we carried out flow cytometry-based immunophenotyping of the major immune population in WS treated vs non-treated group. In line with the previously published reports, our data from lymph-node cells shows that intranasal SARS-CoV-2 infection causes severe lymphopenia in hACE2.Tg mice were characterized by a significant decrease in lymphocytes (CD45+), total T cells (CD3+) cells, T helper cells (CD4+), and cytotoxic T (CD8+) cells (**Figures 7A–C**). This skewed immune profile was rescued in the RDV group. WS group also showed recovery in depleted CD45+ cells but failed to show any significant recovery in T cell frequency. In addition to T cell depletion, COVID-19 is also characterized by a high frequency of inflammatory monocytes and myeloid-derived suppressor cells (MDSCs) in clinical cases (58). In line with this, we found a high frequency of monocytes in the lymph nodes in I control which was significantly reduced in WS or RDV-treated group. Moreover, the high levels of MDSCs were significantly suppressed in both WS and RDV groups. However, we did not find any observable difference in the percentage frequency of NK, NKT, macrophages, or neutrophils (**Figures 7D–E**). Taken together, we found that WS-treated mice show a rescuing effect in the dysregulated immune profile following SARS-CoV-2 infection.

### WS effectively inhibits inflammatory cytokine in hACE2.Tg mice

**Figure 8:**
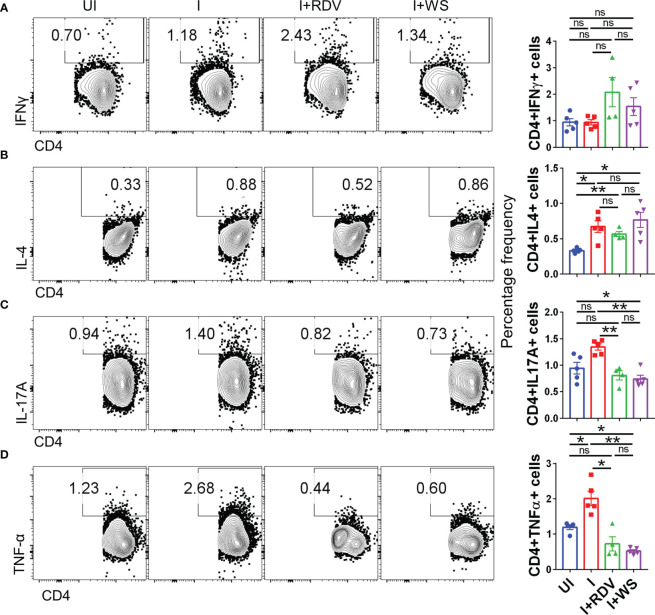
Changes in the effector cytokines of CD4+ T cells of infected hACE2 mice with or without treatment. Flow cytometry-based quantitation was done to evaluate changes in the major immune population in the lymph nodes of sacrificed animals at 6 dpi. The % age frequency was plotted as bar graph along with the representative contour plot **(A)** CD4+IFNγ+ cells **(B)** CD4+IL4+ cells **(C)** CD4+IL17A+ cells **(D)** CD4+TNFα+ cells for each experiment N=5. One way-Anova using non-parametric Kruskal-Wallis test for multiple comparison. *P < 0.05, **P < 0.01.

In order to understand the immunomodulatory potential of WS in the *in-vivo* settings, we performed intracellular cytokine staining (ICS) of phorbol myristate acetate (PMA), Ionomycin-activated lymph-node cells isolated from challenged hACE2.Tg mice. Based on the results obtained from ICS, we did not find any difference in Th1 (CD4+IFNγ+) and Th2 (CD4+IL-4+) response in the WS group, however, there was 2-3-folds inhibition of Th17 cells (CD4+IL17A+) and TNFα secreting CD4+ T cells in WS group as compared to the I control (**Figures 8A–D**). We also studied the effector cytokine response in CD8+ T cells and NK cells compartment. Effector cytotoxic response is correlated with improved survival and morbidity in COVID-19 cases, while NK cell activity is crucial for early viral clearance and immunity. Our data shows WS did not significantly modulate the cytokine profile of CD8+ and NK cells, when compared to the I control profile (**Supplementary Figures S5A–G**). However, we did see significant inhibition in the CD8+IFNγ+ T cell response in presence of WS *in-vivo*. Taken together, CD4+ T cell-specific inhibition was mediated by WS *in-vivo*, while cytokine response in the cytotoxic T cell compartment was relatively unaltered.

### WS mitigates COVID-19 pathology in the animal model by anti-inflammatory response

**Figure 9:**
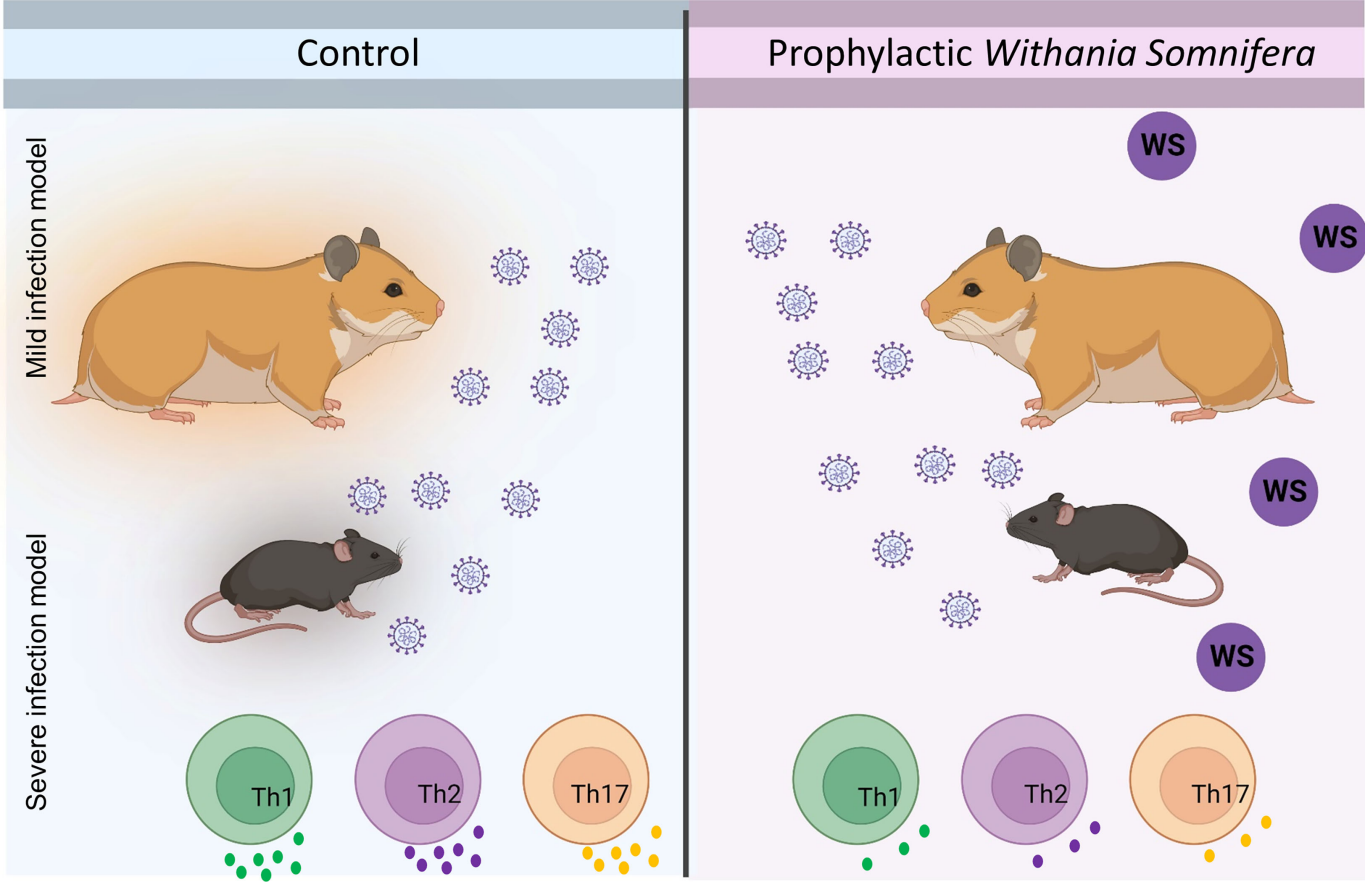
Summary figure highlighting the study design and novel findings from the study.

In summary, we show by using two animal models viz hamster and hACE2.Tg mice that animals receiving prophylactic treatment of WS were broadly protected against COVID-19 both in terms of pulmonary pathology and lung viral load. This protection was further shown to be associated with immunosuppressive activity by WS, especially in the CD4+ T cells effector response both *in-vitro* and *in-vivo* (**Figure 9**).

## Discussion

Since its first reported case, COVID-19 has led to an unprecedented number of clinical cases and mortality warranting urgent prophylactic and therapeutic interventions. The vaccination strategy was found to be largely useful against the SARS-CoV-2 ancestral strain, however, the subsequent rise in mutant strains led to immune evasion and decreased efficacy of vaccines (13, 14). So far only a few therapeutic drugs have been approved by FDA for COVID-19, while new drugs based on chemical entities require relatively more time for development and are also associated with off-target or safety concerns (5). Interventions based on time-tested herbal extracts have offered an exciting alternate strategy due to their prolonged human use, acceptance, and safety as well as efficacy against infectious diseases (19, 59).

In the current study, we used well-characterized extracts of WS and TC two commonly used Asian traditional medicines to investigate their protective effect against COVID-19 by using hamster and hACE2.Tg mice model. WS contains a large number of phytoconstituents including steroids, alkaloids, saponins, glycosides, volatile oils, sitoindosides, and others with various pharmacological activities (60). Various chemical constituents such as diterpenoid lactones, glycosides, steroids, sesquiterpenoid, phenolics, aliphatic compounds, essential oils, fatty acids, and polysaccharides are present in TC with known biological activities (61)

Various groups have shown that WS constituents could act as a potent inhibitor of SARS-CoV-2. *In silico* studies have shown that Withanone interacts and blocks the activity of both spike glycoprotein and 3CLpro protease which are crucial for virus entry and replication (62). Furthermore, the detailed interactive sites of Withanone and its non-covalent interactions were also elucidated suggesting that Withanone/WS could exhibit protective efficacy *in-vivo* (26). Withaferin A, which is another Withanolide derived from WS, has also been shown to possess anti-viral and immunomodulatory activity as per the *in-silico* studies (26). A detailed characterization of the active ingredients of WS along with other herbal extracts was reported recently by our group, in which the *in-vitro* anti-viral activity of WS extract components was shown for the first time (28). On the other hand, TC, another important herbal extract, has also been previously used and shown to have anti-viral potential through docking studies (25). Though there existed *in-silico* evidence supporting the rationale that WS and TC may be helpful drug candidates for anti-viral activity against SARS-CoV-2, these observations lacked experimental animal model efficacy studies. Moreover, a recently published report by Kataria S et al, 2022 found that Ayurvedic formulation containing TC and PL in addition to the first line of treatment for COVID-19 was beneficial in reducing the duration of hospitalization and time of recovery for COVID-19 patients warranting studies aimed at evaluating the combinatorial effect of TC and PL in the *in-vivo* model. However, the immunological correlates of protection elicited by WS or TC, if any, against COVID-19 have also been investigated.

In the hamster COVID-19 model, WS showed robust rescuing in the loss of body weight and pulmonary pathologies which were comparable to the RDV group validating the protective efficacy of WS. Notably, the WS group showed a 7-8 folds decrease in the lung viral load of infected hamsters while no protection was seen in other groups viz TC and TC+PL. It is, therefore, reasonable to argue that WS could inhibit the SARS-CoV-2 entry by blocking its interactive sites as shown previously through *in-silico* studies. However, *in-vitro* validation of the anti-viral potential of WS warrants further examination. Moreover, the decrease in lung viral load also led to the overall recovery of the pulmonary pathology in the WS group. The immunomodulatory potential of Withanolides has been well documented and has been shown to promote an anti-inflammatory environment (22). In line with this, we found that WS-treated hamsters exhibited a significant reduction in the mRNA expression of pro-inflammatory cytokines and boosted the expression of anti-inflammatory cytokines and transcription factors in the hamsters infected with SARS-CoV-2. Since the inflammatory response has been implicated in pulmonary pathology, increased risk of hospitalization and mortality, it could be argued that the anti-inflammatory potential of WS could be the basis of protection together with its anti-viral activity. This was an important finding which showed in parts that WS in COVID-19 led to immunomodulatory effects beneficial for recovery.

The other major arm of immunity that plays a key role in the COVID-19 protective response is the adaptive immune response mediated by T & B cells. Due to a lack of antibody resources for the hamsters to study cellular immunological response, we used hACE2.Tg mice to study the immunological response following infection and the effect of WS (32, 56). Our hACE2.Tg mice data gave two crucial insights, it showed that the WS group may not be fully protected in severe COVID-19 cases as infected hACE2.Tg mice though marginally protected ultimately died following SARS-CoV-2 infection. It is likely that the WS-mediated SARS-CoV-2 inhibition through spike interaction and other entry protease inhibition might not be sufficient to prevent the viral entry and multiplication into the hACE2-expressing lung epithelial cells and therefore might allow virus entry and replication leading to disease pathology. However, notably, the overall pulmonary pathology as examined by histopathological assessment did show significantly less lung injury. Two, immunophenotyping data from hACE2.Tg mice showed recovery from the lymphopenia and dysregulated immune profile in the WS group. Interestingly, we found that the effector cytokine response specific to Th cells was specifically inhibited in presence of WS both *in-vitro* and *in-vivo*. Though, WS exhibited inhibition of the differentiation of Th1, Th2, and Th17 cells *in-vitro*, in the infected hACE2.Tg mice WS treated only showed significant inhibition in Th17 and TNFα secreting CD4+ T cells. Notably, heightened TNFα levels have been correlated with a higher risk of mortality in COVID-19 cases and were associated with cellular injury. Since WS inhibitory potential was specific for CD4+ T cells and not CD8+ T cells or NK cells it is a possibility that WS may be interfering with Th cell activation signaling or downstream signaling involved in the effector response. In addition, WS was also found to suppress the frequency of MDSCs and monocytes which have been shown to promote COVID-19 severity. It is a possibility that the immunomodulatory effect of WS could be acting through both innate and adaptive arm of immunity. Extensive and meticulously designed studies are needed to investigate further the molecular basis for this specificity.

In the infected mice, we did not find any modulation in the neutrophil number. In infected human subjects, however, the neutrophil number is enhanced and NETosis has been linked with thrombosis in COVID-19 pathologies. We, therefore, performed *in vitro* studies on mice BMDNs and human neutrophils. To further look into the possible mechanism of protection, we carried out a detailed investigation of immunological changes. In one part of the study, TRLM primed and PMA and calcium ionophores-induced neutrophil functions were studied in the presence of WS and TC. The ROS generation pathways in the neutrophils have been classically mediated either by NOX2-dependent *via* the activation of protein kinase C (63, 64) or NOX2-independent by calcium-activated small conductance potassium channel (SK3) and/or non-selective mitochondrial permeability transition pore (mPTP) in inducing mtROS production *via* intracellular Ca^2+^ flux (65, 66) Additionally, many reports had also postulated the crosstalk of NOX2 activation and mtROS production and thus represents a feed-forward vicious cycle for ROS generation in oxidative stress (67). By using mitochondrial-targeted inhibitors of NOX2, Vorobjeva et al. (2020) showed the involvement of both NOX2-derived ROS and mtROS in NETosis in human PMNs (66). We also observed a large amount of ROS and mtROS production induced by PMA and/or ionophores. WS and TC have been known to contain anti-oxidants with potent free-radical scavenging abilities. We found a significant reduction in the oxidative stress in TC and WS pretreated cells as demonstrated by their ability to suppress both ROS and mtROS. However, the anti-oxidant properties of both WS and TC were comparatively more pronounced in the case of PMA than ionophores. This indicates a putative role of WS and TC primarily as the NOX2-targeted effector, however, the inhibitory effect of these herbal extracts on the mtROS – NOX2 feed-forward cycle cannot be omitted.

NETosis is a distinct process of cell death unlike necrosis, apoptosis, or necroptosis (50); the molecular processes involved in NETosis are now better understood (66, 68). NETs are characterized by initial morphological changes and histone modifications followed by mechanical changes leading to chromatin decondensation and an irreversible rupture of nuclear and cell envelope (Neubert et al., 2018). Moreover, Awasthi et al. (2016) had reported the involvement of TLRs in NETosis; they have found that blocking TLR-2 and -6 with specific antibodies could significantly inhibit oxLDL induced NETosis (69). In agreement with this, we have also found a notable reduction in NETs formation in TC pre-treated cells in response to TRLM primed and PMA/calcium ionophores activation. We found that TC predominantly inhibited neutrophil NOX-2 activity as evidenced by immunolabeling of MPO and citrullinated histones. NOX-2 is the most abundant protein and an important source of superoxide radicals in neutrophils that mediate PMA-induced NETosis. Since TC preferentially inhibited ROS, mtROS, and NETs production largely stimulated by PMA, we postulate that TC antagonizes an upstream component at the early stage of NOX2-mediated NETosis.

Neutrophils are the first phagocytic cells to reach the site of infection or injury (70). WS and TC did not reveal any significant adversity on phagocytosis by neutrophils when these were pre-treated with up to 300 µg/ml concentrations of the extracts; however, a low of 20% was observed with TC. Moreover, the intracellular bactericidal capacity of neutrophils also did not show any notable change with the extracts. Together, these results suggested that modulation of neutrophil functionality could be one of the contributing factors for WS-mediated protection against COVID-19.

## Future prospects

Our results provide the first direct evidence that prophylactic WS administration is affecting in mitigating the pathology of COVID-19 which is mediated by the anti-inflammatory potential of WS through suppression of effector T helper cell response. Though there is pool of literature available based on computational analysis as well as *in-vitro* activity suggesting the active pharmaceutical ingredients of WS which may be essential for these immunomodulatory potentials, but lacks data from animal studies. Future experiments should be designed to investigate and decipher the therapeutically potential of WS ingredient and to optimize its dosage which could be taken for randomized clinical trials. Moreover, the pharmacological potential of WS in combination with COVID-19 anti-viral drug or vaccine candidates could be exploited to better understand the synergistic effect of the treatment. In addition, future proof of concept studies could be designed for other infectious diseases which are known to cause cytokine release syndrome such as Influenza, to test if it helps mitigate the disease.

## Conclusion

Though ancient knowledge of medicinal potential of herbs existed in Ayurvedic science since long we did not have much scientific evidence about its protective/preventative efficacy from animal studies and more so on COVID19. In this study, by combining hamster and hACE2 transgenic mice model we provide direct evidence that prophylactic treatment of WS mitigates COVID19 through its anti-inflammatory properties. Our findings are important in the context of a continuously evolving virus that leads to immune evasion by previous vaccination and warrants a more robust therapeutic approach against emerging variants of SARS-CoV-2. We also defined the immunological correlates of protection based on *in-vitro* and *in-vivo* studies and believe that the potent anti-inflammatory potential of WS could be further exploited against other infectious diseases and inflammatory disorders (one such clinical trial, CTRI/2021/06/034496, for this is already conducted in India for WS). Finally, our study supports the use of WS to prevent COVID-19 pathologies and may also be evaluated for its efficacy against other viral infections.

## Data Availability

The original contributions presented in the study are included in the article/**Supplementary Material**. Further inquiries can be directed to the corresponding authors.

## Supplementary material

Supplementary Figure 1 Effect of WS and TC on PMA/A23187 induced cytosolic ROS and mtROS production in mice BMDNs. BMDNs pre-incubated at different concentrations of TC and WS were stimulated with optimal concentration of PMA (100 nM) and A23187 (10 µM) for 30 min. DCF-DA (10 µM) and MitoSOX (10 µM) were used for cytosolic ROS and mtROS detection, respectively using flow cytometry. BMDNs pre-incubated at different concentrations of TC and WS were stimulated with optimal concentration of PMA (100 nM) and A23187 (10 µM) for 30 min. SYTOX Green (100 nM) was used to monitor extracellular DNA release using a plate reader (**A, B**: TC; **C, D**: WS). Total MFI in each experimental condition is expressed as Mean ± SEM of min 3 experiments. All the data are represented as Mean ± SEM, n = min 3 per group, and statistical analysis consisted of one-way ANOVA followed by Bonferroni’s test (*p < 0.05, **p < 0.01, ***p < 0.001 vs respective control groups; #p<0.05, ##p<0.01 vs PMA/A23187 treated groups). C, control; N, N-acetyl cysteine; MT, MitoTEMPO. C, control; V, VAS2870; D, Diltiazem. Cytotoxic potential of WS and TC on human PMNs and murine BMDNs. Percent cell death was obtained by flow cytometry using PI (10 µg/ml). Doxorubicin (10 µM) was used as a positive control (100%).

Supplementary Figure 2 Effect of WS and TC on phagocytosis and bactericidal activity of human PMNs. **(A, B)** Human PMNs were incubated with different concentrations of WS and TC before adding PE-labelled latex beads for phagocytic assay. Fluorescent signal was quenched using trypan blue (0.4%) before acquiring in FACS cell analyzer. **(C, D)** Effect of GG on bactericidal activity of human PMNs. Cells were pre-treated with 300 µg/ml of WS and TC before incubating with kanamycin-resistant *E. coli*. The direct effect of WS and TC on *E. coli* growth was also monitored by incubating bacteria with 300 ug/ml concentration for 30 min. **p < 0.01 vs control groups; ##p<0.01 vs GG treated groups. All the data are represented as Mean ± SEM, n = min 3 per group, and statistical analysis consisted of one-way ANOVA followed by Bonferroni’s test. C, control; WS300, WS 300 μg/ml; TC300, TC 300 μg/ml.

Supplementary Figure 3 Immunosuppressive effect of *Tinospora cordifolia* (Willd.) Miers on *in vitro* differentiation of Th1, Th2 and Th17 cells. Naïve CD4+ T cells were sorted from mice spleen and lymph nodes and were activated by soluble anti-CD3 antibody. Cells were then differentiated into helper T (Th) 2 **(A, B)**, Th17 cells **(D, E)** and Th1 conditions **(G, H)** using recombinant mouse IL-4; TGF-β + IL-6 and IL-12 cytokines respectively in presence or absence of dexamethasone (doses ranging from 1pM to 20uM). Differentiated cells at 72 hours were quantitated by performing intracellular cytokine staining for IL-4, Il-17 and IFN-gamma production. IC50 values were calculated using Graph pad prism software **(C, F, I)**. *P < 0.05, **P < 0.01, ***P < 0.001, ****P < 0.0001 by one-way ANOVA.

Supplementary Figure 4 Immunosuppressive effect of Dexamethasone on *in vitro* differentiation of Th1, Th2 and Th17 cells. Naïve CD4+ T cells were sorted from mice spleen and lymph nodes and were activated by soluble anti-CD3 antibody. Cells were then differentiated into helper T (Th) 2 **(A, B)**, Th17 cells **(D, E)** and Th1 conditions **(G, H)** using recombinant mouse IL-4; TGF-β + IL-6 and IL-12 cytokines respectively in presence or absence of dexamethasone (doses ranging from 1pM to 20uM). Differentiated cells at 72 hours were quantitated by performing intracellular cytokine staining for IL-4, Il-17 and IFN-gamma production. IC50 values were calculated using Graph pad prism software **(C, F, I)**. *P < 0.05, **P < 0.01, ***P < 0.001, ****P < 0.0001 by one-way ANOVA.

Supplementary Figure 5 Changes in the effector cytokines of CD8+ T and NK cells of infected hACE2 mice with or without treatment. Flow cytometry-based quantitation was done to evaluate changes in the major immune population in the lymph nodes of sacrificed animals at 6 dpi. The % age frequency was plotted as bar graph along with the representative contour plot **(A)** CD8+Gzb+ cells **(B)** CD8+Prf-1+ cells **(C)** CD8+TNFα+ cells **(D)** CD8+IFNγ+ cells **(E)** CD8+IL4+ cells **(F)** CD8+IL17A+ cells **(G)** CD3-NK1.1+ IFNγ+ cell population. Granzyme (Gzb), Perforin (Prf-1). For each experiment N=5. One way-Anova using non-parametric Kruskal-Wallis test for multiple comparisons. *P < 0.05, **P < 0.01.

## Glossary

**Table:**

ABSL3	animal biosafety level 3
COVID-19	coronavirus disease 19, set of clinical pathologies following of coronavirus infection
CRS	cytokine release syndrome
DXM	Dexamethasone
FACS	floourescence activated cell sorting
hACE2 mice	Transgenic mice developed by knocking in humanized ACE2 receptors under the influence of the K18 promoter
Hamster	golden Syrian hamster, the animal model used for COVID-19 study
Herbal extract	Aqueous Extract of herbs that have been used in the study
I	Infected
I+TC	infected animals receiving TC
I+TC+PL	infected animals receiving TC in combination with PL
I+WS	infected animals receiving WS
IFNγ	interferon gamma
IL6	interleukin 6
mtROS	ROS generated in mitochondria
Muc-1	mucin 1 gene
NET	neutrophil extracellular traps
Pathology	clinical features of the disease that can be used for diagnosis and examination
Pfu	plaque forming unit
PL	*Piper longum* (L.)
qPCR	quantitative PCR
RDV	remdesivir
ROS	reactive oxygen species
SARS-CoV-2	severe acute respiratory syndrome coronavirus 2, the virus responsible for COVID-19.
Sftp-D	surfactant protein D
T helper cells	component of cell-mediated immunity which express CD4 marker
TC	*Tinospora cordifolia* (Willd.) Miers
Th1 cells	Subset of CD4+ T helper cells which are important for anti-viral immunity
Th17 cell	subset of CD4+ T helper cells which are important of inflammation and autoimmunity
Th2 cells	subset of CD4+ T helper cells which are important for extra cellular pathogens
TNFα	tumor necrosis factor alpha
UI	uninfected
WS	*Withania somnifera* (L.) Dunal
