# Corrigendum: Pharmacological potential of *Withania somnifera* (L.) Dunal and *Tinospora cordifolia *(Willd.) Miers on the experimental models of COVID-19, T cell differentiation, and neutrophil functions

**DOI:** 10.3389/fimmu.2025.1594972

**Published:** 2025-05-05

**Authors:** Zaigham Abbas Rizvi, Prabhakar Babele, Upasna Madan, Srikanth Sadhu, Manas Ranjan Tripathy, Sandeep Goswami, Shailendra Mani, Madhu Dikshit, Amit Awasthi

**Affiliations:** ^1^ Immuno-biology Lab, Translational Health Science and Technology Institute, NCR-Biotech Science Cluster, Faridabad, Haryana, India; ^2^ Immunology-Core Lab, Translational Health Science and Technology Institute, NCR-Biotech Science Cluster, Faridabad, Haryana, India; ^3^ NCD, Translational Health Science and Technology Institute (THSTI), NCR Biotech Science Cluster, Faridabad, Haryana, India; ^4^ Pharmacology, CSIR-Central Drug Research Institute, Lucknow, Uttar Pradesh, India

**Keywords:** *Withania somnifera (Ashwagandha)*, *Tinospora cordifolia*, SARS-CoV-2, hamster model, T cells, neutrophils, and hACE2 transgenic mice, COVID-19

In the published article, there was an error in **Figures 5A** and **5D** (FACS panel of Th0 & Th1; and Th0 & Th2) as published. The FACS images in **Figures 5A** and **5D** (Th0 & Th2 panel) were originally generated by Upasna Madan, a graduate student, as part of a broader project investigating the *in vitro* immunomodulatory effects of eight herbal extracts. Initially, all experiments were conducted with multiple biological and technical replicates, intending to publish the findings in a single manuscript. However, during analysis, we determined that presenting the data in multiple manuscripts, each focusing on a specific herbal extract, would be more effective.

**Figure 5:**
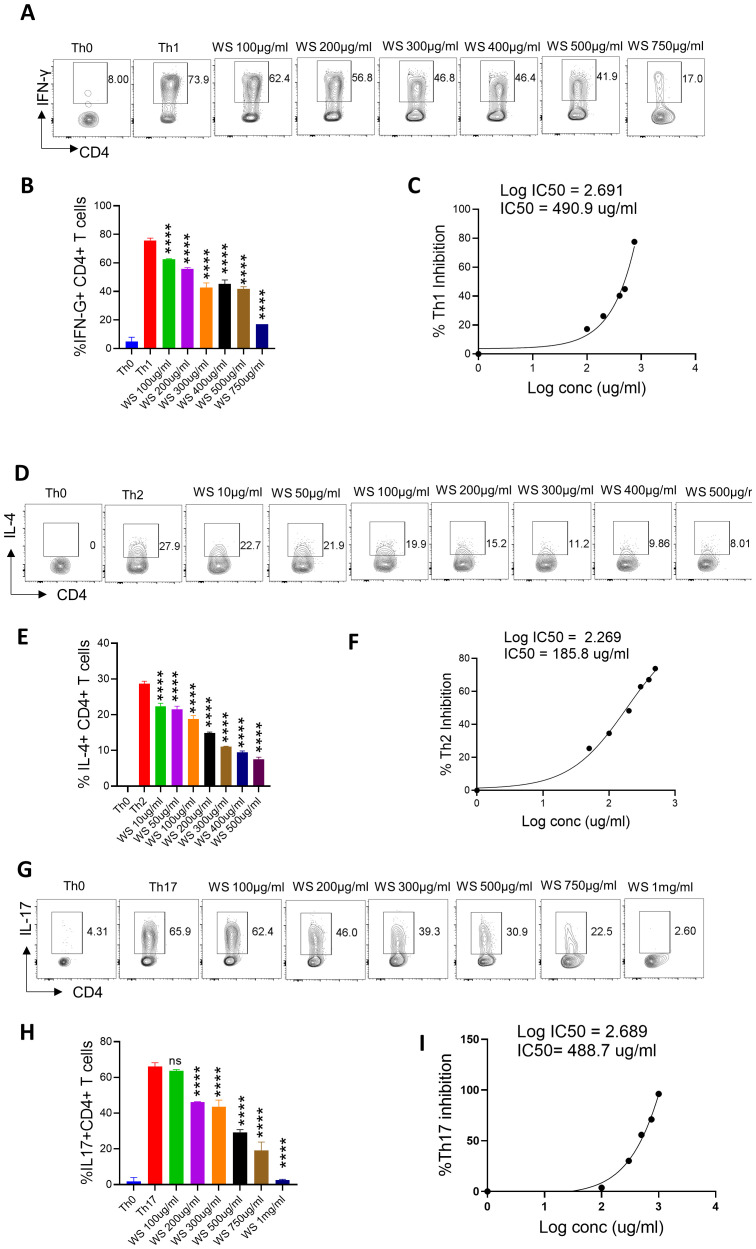
Dose kinetics of WS response on *in vitro* differentiation of Th1, Th2 and Th17 cells from naïve CD4+ T cells. Sorted naïve CD4+ T cells from mouse spleen and lymph nodes were activated using soluble anti-CD3 antibody and differentiated into helper T (Th)2 **(A, B)**, Th17 cells **(D, E)** and Th1 subtypes **(G, H)** by using different cytokines viz recombinant mouse IL-4; TGF-β + IL-6 and IL-12 cytokines respectively. WS was added in concentrations ranging from 10ug/ml to 1000ug/ml initially at the time of cell seeding. After 72 h of incubation IL-4, Il-17 and IFN-gamma production was measured respectively for Th2, Th17 and Th1 cells by intracellular cytokine staining. IC50 values were calculated using Graph pad prism software **(C, F, I)**. ****P < 0.0001 by one-way ANOVA.

During this segmentation process, representative control images from the FACS analysis were inadvertently reused in two different manuscripts (PMID: 36960064 & PMID: 37765142). Since each experiment included multiple control replicates, we are in the process of replacing the affected control FACS plots. Importantly, as these FACS plots pertain only to control conditions, their replacement does not alter the overall findings, interpretations, or conclusions of either manuscript.

The corrected **Figures 5A** and **5D**, Th0 & Th2 panel and its caption appear below.

The authors regret this oversight and affirm that it does not impact the scientific conclusions of the article. The original article has been updated accordingly.

