# Supplementary figures and images for: Pharmacological potential of Withania somnifera (L.) Dunal and Tinospora cordifolia (Willd.) Miers on the experimental models of COVID-19, T cell differentiation, and neutrophil functions
Source: Front Immunol. 2023 Mar 7;14:1138215. doi: 10.3389/fimmu.2023.1138215 (PMC10028191; doi:10.3389/fimmu.2023.1138215)

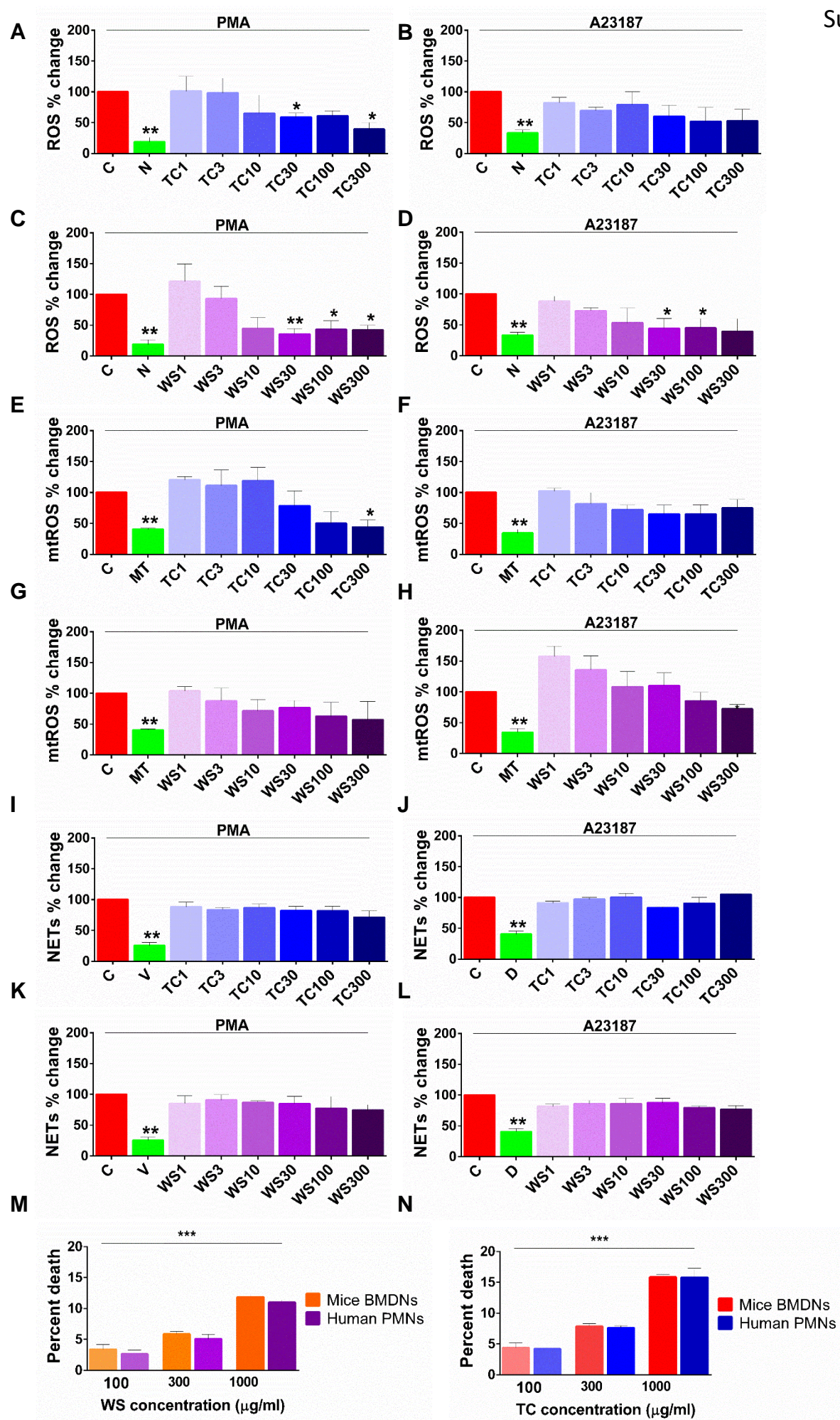

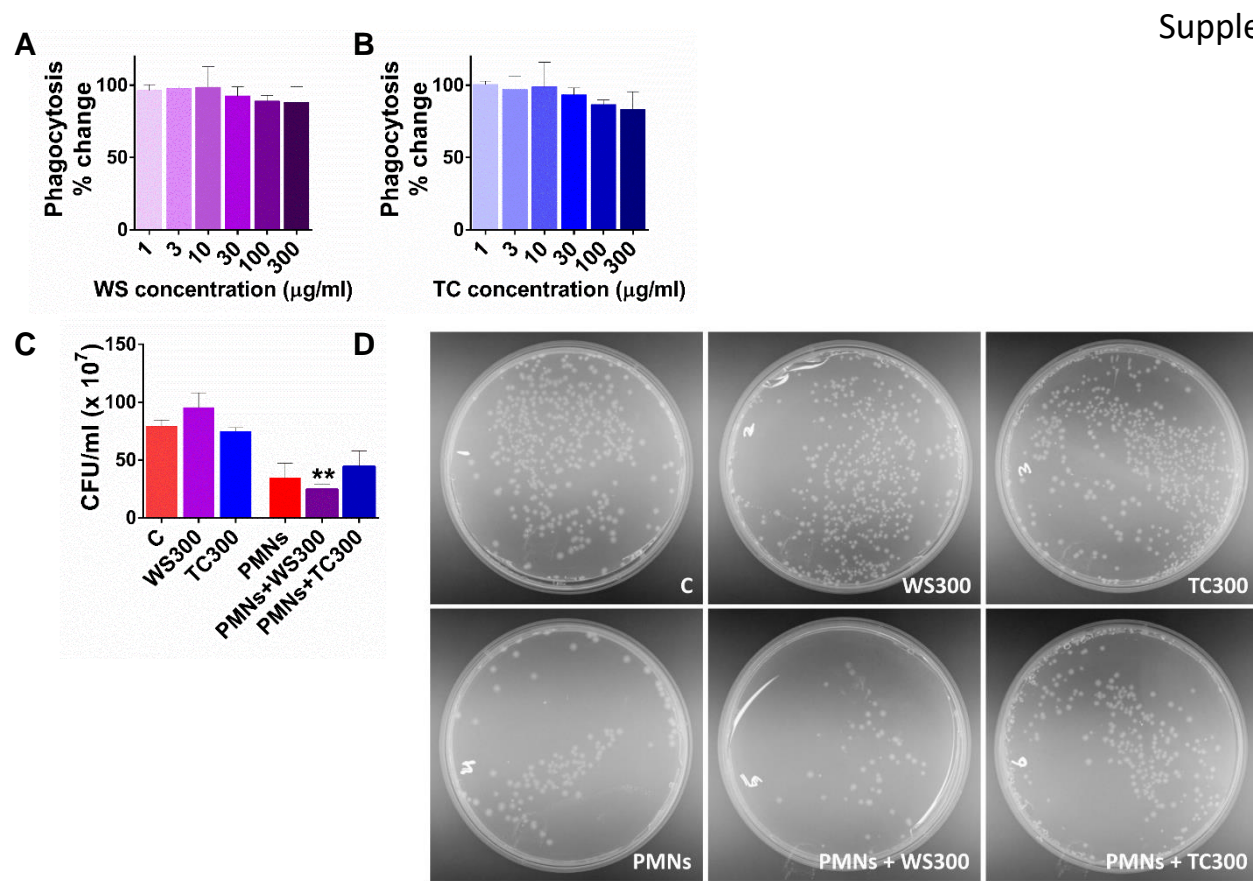

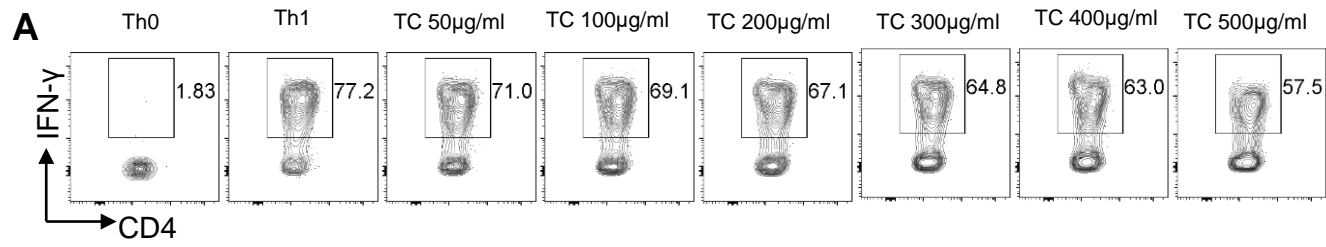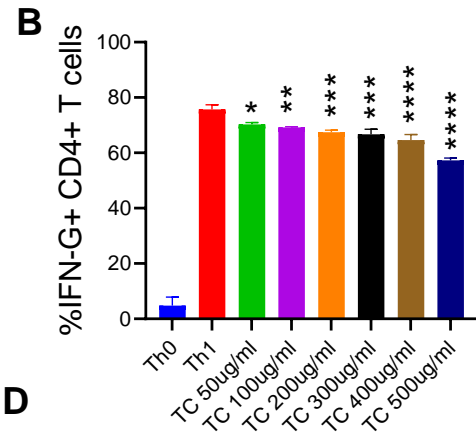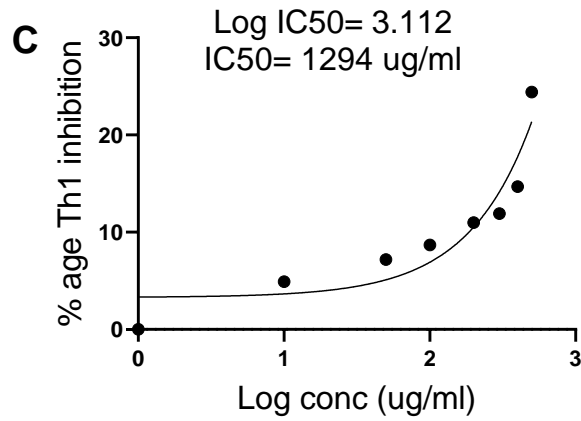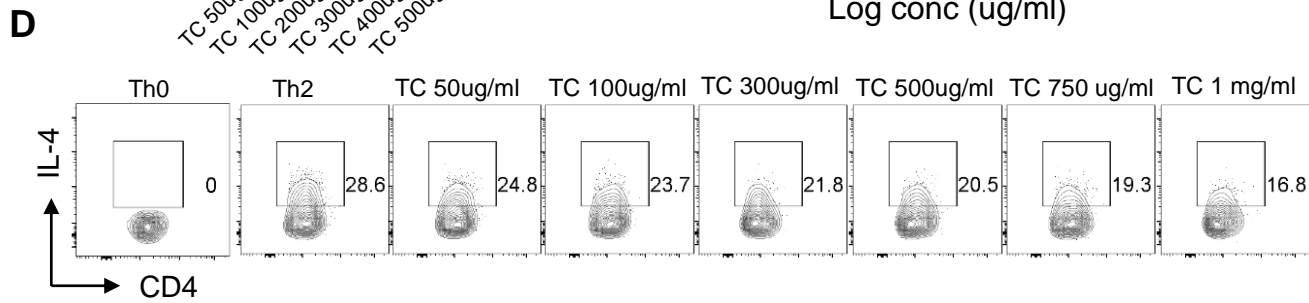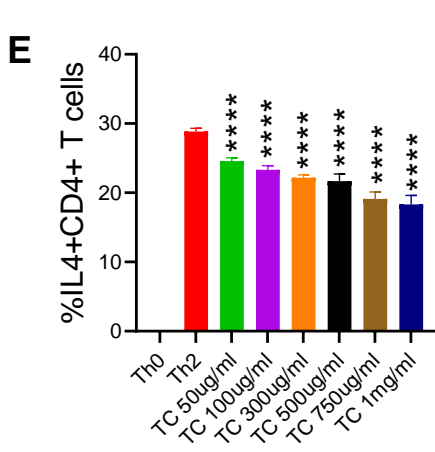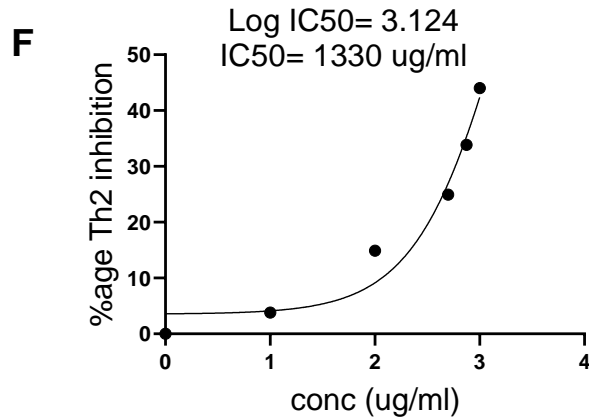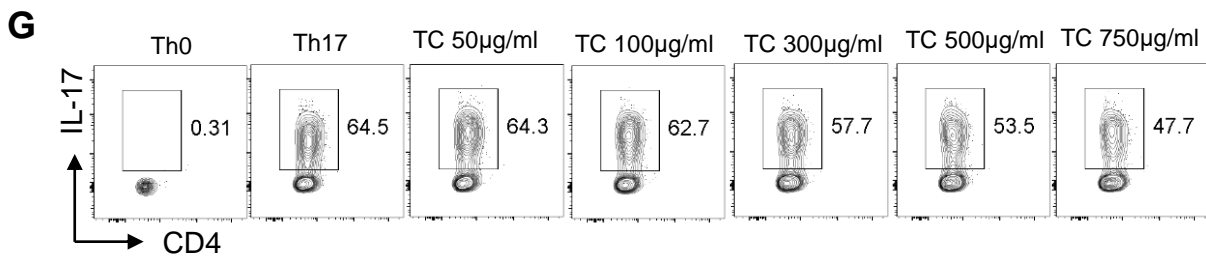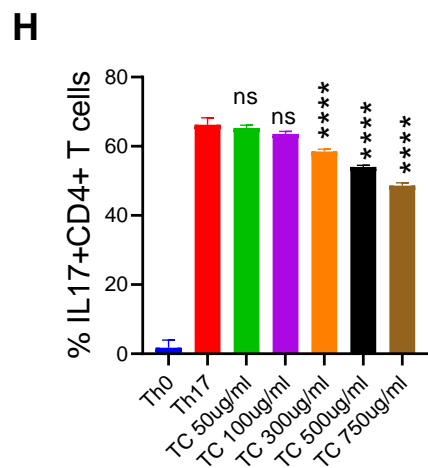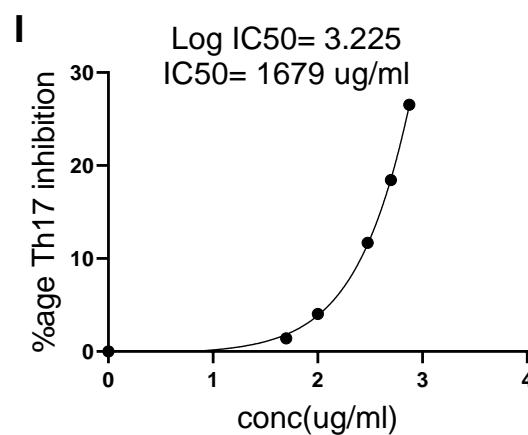

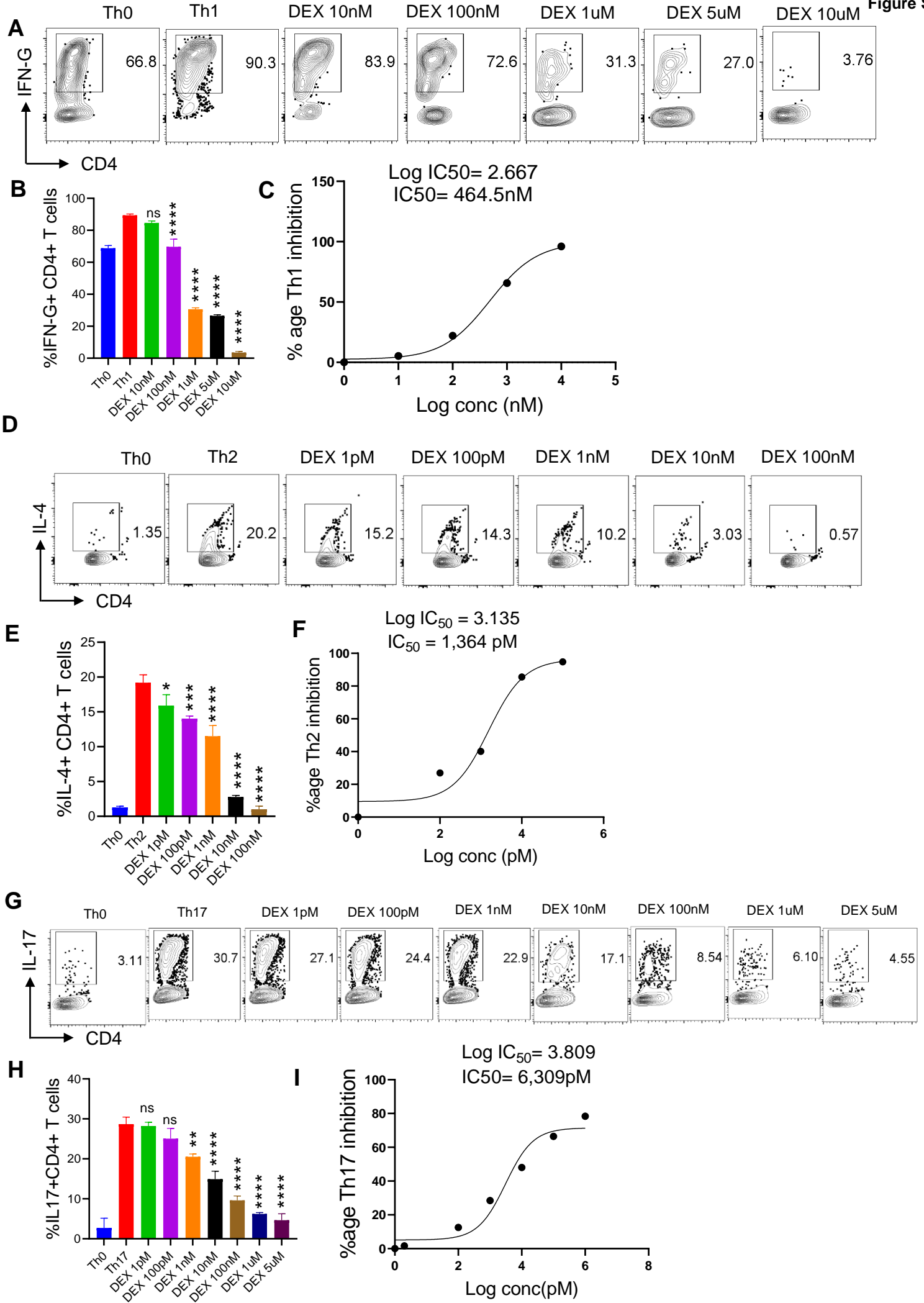

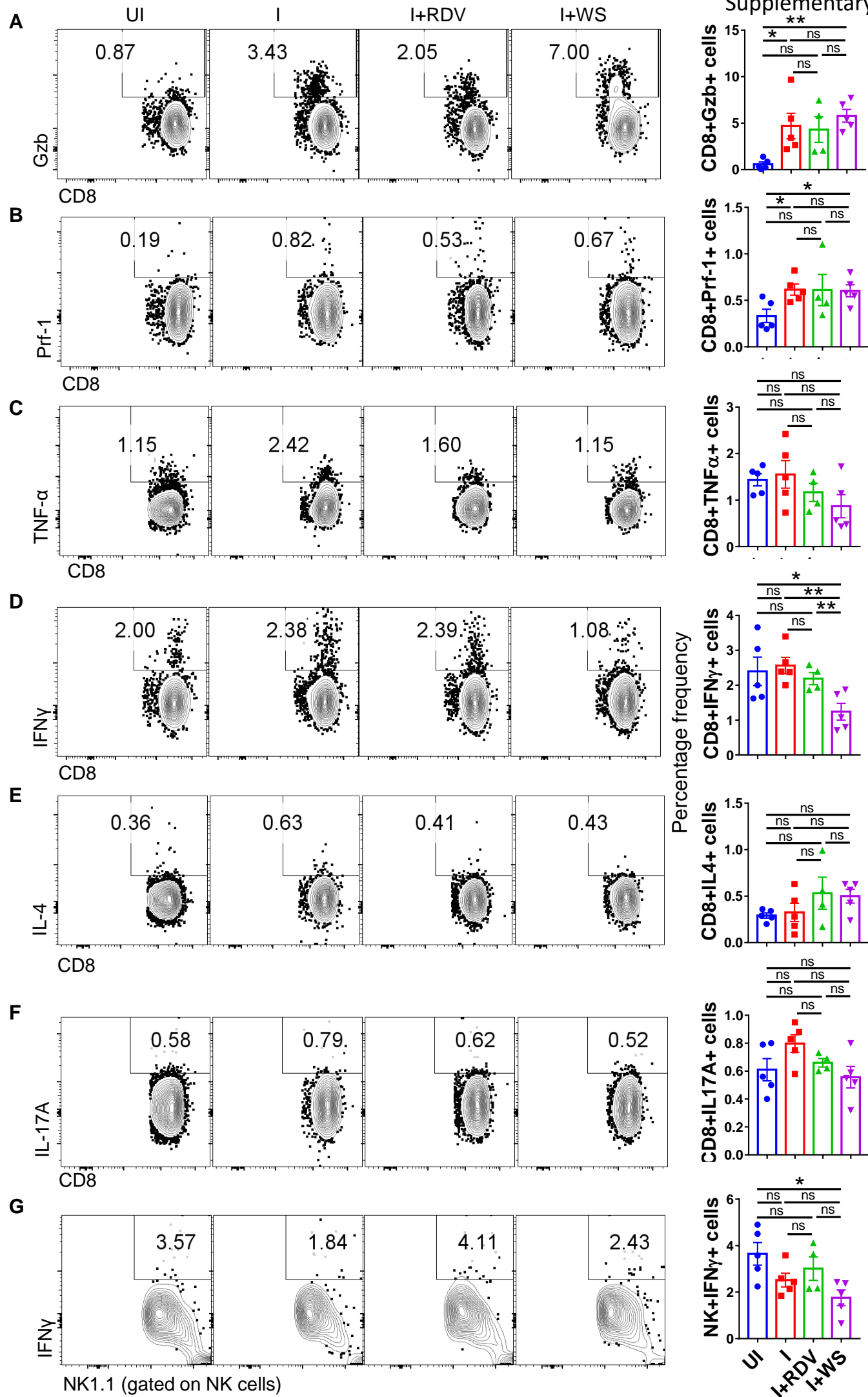

Supplement: Supplementary Figure 1 — Effect of WS and TC on PMA/A23187 induced cytosolic ROS and mtROS production in mice BMDNs. BMDNs pre-incubated at different concentrations of TC and WS were stimulated with optimal concentration of PMA (100 nM) and A23187 (10 µM) for 30 min. DCF-DA (10 µM) and MitoSOX (10 µM) were used for cytosolic ROS and mtROS detection, respectively using flow cytometry. BMDNs pre-incubated at different concentrations of TC and WS were stimulated with optimal concentration of PMA (100 nM) and A23187 (10 µM) for 30 min. SYTOX Green (100 nM) was used to monitor extracellular DNA release using a plate reader (A, B: TC; C, D: WS). Total MFI in each experimental condition is expressed as Mean ± SEM of min 3 experiments. All the data are represented as Mean ± SEM, n = min 3 per group, and statistical analysis consisted of one-way ANOVA followed by Bonferroni’s test (*p < 0.05, **p < 0.01, ***p < 0.001 vs respective control groups; #p<0.05, ##p<0.01 vs PMA/A23187 treated groups). C, control; N, N-acetyl cysteine; MT, MitoTEMPO. C, control; V, VAS2870; D, Diltiazem. Cytotoxic potential of WS and TC on human PMNs and murine BMDNs. Percent cell death was obtained by flow cytometry using PI (10 µg/ml). Doxorubicin (10 µM) was used as a positive control (100%). [file DataSheet1.pdf]
